# A holistic phylogeny of the coronin gene family reveals an ancient origin of the tandem-coronin, defines a new subfamily, and predicts protein function

**DOI:** 10.1186/1471-2148-11-268

**Published:** 2011-09-25

**Authors:** Christian Eckert, Björn Hammesfahr, Martin Kollmar

**Affiliations:** 1 Department of NMR-based Structural Biology, Max-Planck-Institute for Biophysical Chemistry, Am Fassberg 11, 37077 Goettingen, Germany

## Abstract

**Background:**

Coronins belong to the superfamily of the eukaryotic-specific WD40-repeat proteins and play a role in several actin-dependent processes like cytokinesis, cell motility, phagocytosis, and vesicular trafficking. Two major types of coronins are known: First, the short coronins consisting of an N-terminal coronin domain, a unique region and a short coiled-coil region, and secondly the tandem coronins comprising two coronin domains.

**Results:**

723 coronin proteins from 358 species have been identified by analyzing the whole-genome assemblies of all available sequenced eukaryotes (March 2011). The organisms analyzed represent most eukaryotic kingdoms but also cover every taxon several times to provide a better statistical sampling. The phylogenetic tree of the coronin domains based on the Bayesian method is in accordance with the most recent grouping of the major kingdoms of the eukaryotes and also with the grouping of more recently separated branches. Based on this "holistic" approach the coronins group into four classes: class-1 (Type I) and class-2 (Type II) are metazoan/choanoflagellate specific classes, class-3 contains the tandem-coronins (Type III), and the new class-4 represents the coronins fused to villin (Type IV). Short coronins from non-metazoans are equally related to class-1 and class-2 coronins and thus remain unclassified.

**Conclusions:**

The coronin class distribution suggests that the last common eukaryotic ancestor possessed a single and a tandem-coronin, and most probably a class-4 coronin of which homologs have been identified in Excavata and Opisthokonts although most of these species subsequently lost the class-4 homolog. The most ancient short coronin already contained the trimerization motif in the coiled-coil domain.

## Background

The coronin proteins, which were originally isolated as a major co-purifying protein from an actin-myosin-complex of the slime mold *Dictyostelium discoideum *[1], have since been identified in other protists [2,3], fungi [4], and animals [5], but are absent in plants. Coronins are a conserved family of actin binding proteins [6-8] and the first family member had been named coronin based on its strong immunolocalization to the actin rich crown like structures of the cell cortex in *Dictyostelium discoideum *[1]. Coronins belong to the superfamily of the eukaryotic-specific WD40-repeat proteins [9,10] and play a role in several actin-dependent processes like cytokinesis [11], cell motility [11,12], phagocytosis [13,14], and vesicular trafficking [15].

WD-repeat motifs are minimally conserved regions of approximately 40-60 amino acids typically starting with Gly-His (GH) dipeptides 11-24 residues away from the N-terminus and ending with a Trp-Asp (WD) dipeptide at the C-terminus. WD40-repeat proteins, which are characterized by the presence of at least four consecutive WD repeats in the middle of the molecule, fold into beta propeller structures and serve as stable platforms for protein-protein interactions [9].

The coronin proteins have five canonical WD-repeat motifs located centrally. Since the region encoding the WD repeats is similar to the sequence of the beta-subunit of trimeric G-proteins the formation of a five-bladed beta-propeller was assumed for coronins [16]. However, the determination of the structure of murine coronin-1 (*Mm*Coro1A [17]) demonstrated that the protein, analogous to the trimeric G-proteins, forms a seven-bladed beta-propeller carrying two potential F-actin binding sites. Apart from the central WD-repeats, almost all coronin proteins have a C-terminal coiled-coil sequence that mediates homo-oligomerization [18-20], and a short N-terminal motif that contains an important regulatory phosphorylation site in coronin-1B [12]. In addition, each coronin protein has a unique region of variable length and composition following the conserved extension to the C-terminus of the beta-propeller.

Based on their domain composition coronins have originally been divided into two subfamilies, namely short and long coronins [21]. Short coronins consist of 450 - 650 amino acids containing one seven-bladed beta-propeller and a C-terminal coiled-coil region. Furthermore, the N-terminal region of most known short coronins contains 12 basic amino acids. Since this motif is only present in coronin molecules, it has been suggested as a novel coronin signature [21]. The longer types of coronin, also called POD or Coronin 7, possess two complete core domains in tandem but lack a coiled-coil motif. In the longer coronins, the sequence of the basic N-terminal motif is reduced to 5 amino acids. Based on phylogenetic relationships among the coronins, the Human Genome Organization nomenclature committee (HGNC) proposed a system in 2001 that grouped the short coronins into two classes resulting in a total of three subtypes [8]. Very recently, a new nomenclature has been suggested dividing the coronins into twelve subclasses based on the analysis of about 250 coronins from most taxa [22]. In contrast to previous systems, every mammalian coronin (and corresponding vertebrate homologs) was designated an own class resulting in seven vertebrate classes. Invertebrates were grouped into two classes, the fungi got an own class, coronins from alveolates were grouped with those from Parabasalids (class 10), and the remaining coronins from Amoeba, Heterolobosea, and Euglenozoa were combined into the twelfth class. This study constituted the first major phylogenetic analysis of the coronin family. However, this classification was not consistent with the latest phylogeny of the eukaryotes and homologs of some major branches like the stramenopiles were missing.

Here, we present the analysis of the complete coronin repertoires of all eukaryotic organisms sequenced and assembled so far. The distribution of all coronin homologs is in accordance with the latest taxonomy of the eukaryotes and reveals the origin of the tandem-coronin and another newly defined class in the last common ancestor of the eukaryotes.

## Results

### Identification and annotation of the coronin proteins

The coronin protein genes were identified by TBLASTN searches against the corresponding genome data of the different species. The list of sequenced eukaryotic species as well as access information to the corresponding genome data has been obtained from diArk [23]. Species that missed certain orthologs in the first instance were later searched again with supposed-to-be orthologs of other closely related species. In this iterative process all coronin family proteins have been identified or their loss in certain species or taxa was confirmed. Because verified cDNA sequences and protein predictions, which often contain mispredicted exons and introns even in the "annotated" genomes, are not available for most of the sequenced species, the protein sequences were assembled and assigned by manual inspection of the genomic DNA sequences. Exons have been confirmed by the identification of flanking consensus intron-exon splice junction donor and acceptor sequences [24]. In addition, the gene structures of all coronin genes were reconstructed using WebScipio [25,26]. Through comparison of the intron positions and splice-site phases in relation to the protein multiple-sequence alignment, several suspicious exon border predictions could be resolved and the protein sequences subsequently be corrected. The genomic sequences of many species contain several gaps due to the low coverage of the sequencing or problems in the assembly process. Only some of the gaps could be closed at the amino-acid level by analyzing EST data.

The coronin dataset contains 723 sequences from 358 organisms (Table 1). 614 sequences are complete, and an additional 44 sequences are partially complete. Sequences for which a small part is missing (up to 5%) were termed "Partials", while sequences for which a considerable part is missing were termed "Fragments". This difference has been introduced because Partials are not expected to considerably influence the phylogenetic analysis. Several of the genes were termed pseudogenes because they contain too many frame shifts, in-frame stop codons, and missing sequences to be attributed to sequencing or assembly errors.

**Table 1:** Data statistics

	coronin
Sequence	
Total	723
From WGS	700
Domains	7
Amino acids	
Total pseudogenes	7
Pseudogenes without sequence	3
**Completeness**	
Complete	614
Partials	44
Fragments	62
**Species**	
Total	358
WGS-projects	323
EST-projects	112
WGS- and EST-projects	152

### Multiple sequence alignment, phylogenetic analysis, and classification

**Figure 1:**
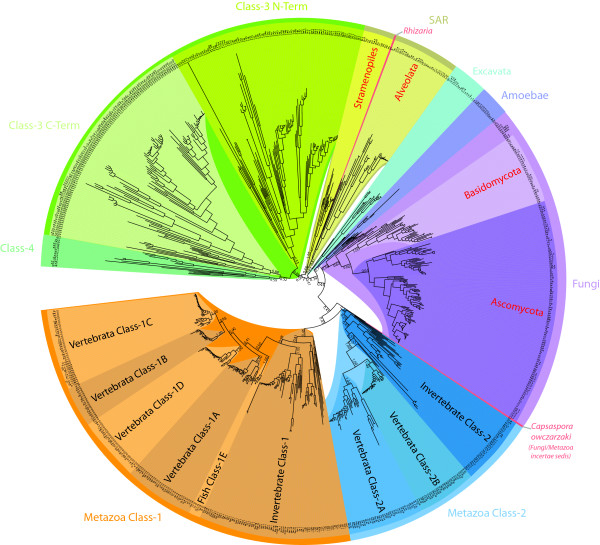
**Phylogenetic tree of the coronin family**. The phylogenetic tree of the coronin family was calculated from the multiple sequence alignment of the conserved coronin domain using the Bayesian method. The unrooted tree was drawn with iTOL [73] and branches were coloured according to class and taxonomic distributions. For an extended representation of the tree including all posterior probability values see Additional file 2.

A multiple sequence alignment of all coronin family members has been created and extensively manually improved (Additional file 1). The basis of the alignment was the conserved coronin domain that consists of the β-propeller region and a subsequent conserved extension, which packs against the "bottom" surface of the propeller [17]. This entire domain is conserved in all coronin homologs and we would therefore suggest naming it coronin-domain. The unique regions following the coronin-domain could only be aligned for homologs of closely related species. The C-terminal predicted coiled-coil regions were aligned again for all corresponding sequences to analyze potential oligomerization patterns (see below). The second coronin-domains of the tandem-coronins were also aligned to the coronin-domains for the phylogenetic analysis. One part of the coronin-domain in coronin-1D is encoded by a cluster of mutually exclusive exons (see below) and therefore the exon with the higher sequence identity to related homologs has been included in the alignment. The phylogenetic tree of the coronin family was calculated for 764 coronin-domains, including both coronin-domains of the tandem-coronins separately, using the Bayesian (Additional file 2) and the maximum-likelihood method (Additional file 3). The resulting trees were almost identical. However, the relations of the innermost nodes representing the most ancient relationships were best resolved using the Bayesian approach (Figure 1). The resulting phylogenetic tree is in accordance with the latest phylogenetic grouping of the six kingdoms of the eukaryotes [27-29] of which five are covered by the data analyzed here. Thus, coronins of phylogenetic related species group together in the coronin family tree. In the coronin tree, not only the grouping is retained but also the evolutionary history of the branches. For example, the fungi separate as monophyletic group before the metazoans, and after the Amoeba.

**Figure 2:**
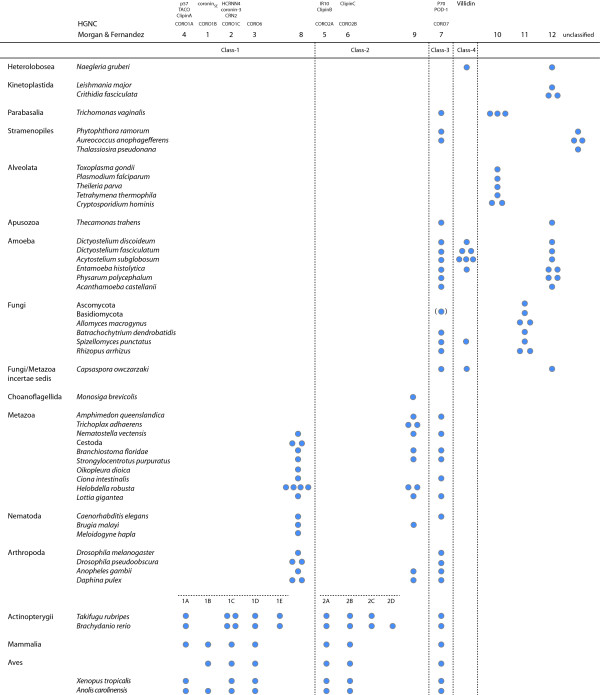
**Coronin repertoire of selected species of major taxa and branches**. The coronins of several representative species for most eukaryotic taxa and branches are listed (for the list of all species see Additional file 4). On top, alternatively used names and classification schemes are given for better comparison and orientation.

The classification into subfamilies should at best include both the phylogenetic grouping of the protein family members and the domain organisation of the respective homologs. However, because most coronins contain a unique region between the coronin-domain and the C-terminal coiled-coil regions, several sub-branch specific domain organisation patterns evolved. To keep the coronin classification as simple as possible and to provide the highest consistency with previous classification schemes, the following classification is proposed: The classification should solely be based on the phylogenetic tree of the coronin-domains because it is in accordance with the phylogeny of the eukaryotes and contains the conserved part of the proteins that is the basis of the protein family. Metazoan species encode two phylogenetically distinct groups of coronins that have historically been named class-1 and class-2 coronins. Further variants of these classes should be named alphabetically, e.g. class-1A, class-1B, etc.. However, due to the independent whole-genome, genomic region, and single gene duplication events of certain phylogenetic branches these variant designations do not always refer to orthologs. For the mammalian coronins, which are the best analyzed coronins, the suggested classification is almost entirely consistent with previous classifications [8] and the HGNC nomenclature except for "CORO6" and "CORO7", which are here classified as coronin-1D and coronin-3, respectively. Class-3 comprises the tandem coronins. All members of this class group together in the phylogenetic tree, and only single homologs have been found in all species analyzed. Class-4 is a newly defined class that contains coronins with variable numbers of C-terminal PH, gelsolin, and VHP domains, but also coronins with only very short sequences outside the coronin-domain. The other coronins group in accordance with the latest taxonomy of the species (Figure 1). In our opinion it does not add information or help the scientific community if those coronins were classified separately. In contrast to the metazoans, gene duplications in the branches of Amoeba, Excavata, and SAR are species-specific and do not warrant further subclassification at the moment. For example, instead of talking about a "class-11 coronin" and long explanations what type of coronins would belong to such a class, it would be easier, shorter, and less confusing to just say a "*Naegleria *coronin", an "apicomplexan coronin" or a "yeast coronin". The distribution of the coronins analyzed here is summarized for some example species in Figure 2 including previously used names and classification schemes. The distribution of all coronins is found in Additional file 4. Coronin homologs are absent in Rhodophyta (*Cyanidioschyzon, Galdieria*), Viridiplantae, Microsporidia, Formicata (*Giardia*), and Haptophyceae (*Emiliania*).

### Short coronins (class-1, class-2, and unclassified coronins)

**Figure 3:**
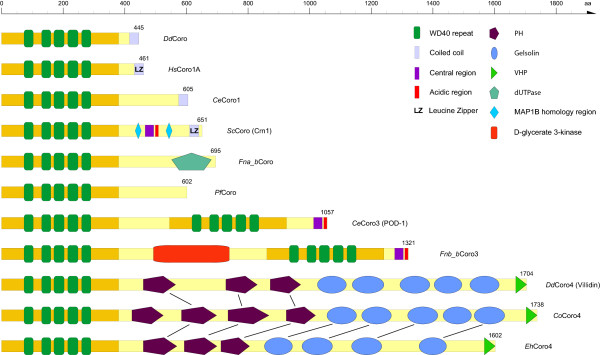
**Domain organisation of representative coronins**. A colour key to the domain names and symbols is given on the right except for the coronin domain that is coloured in orange. The abbreviations for the domains are: WD, WD repeat; PH, pleckstrin-homology domain; LZ, leucine zipper; VHP, villin headpeace domain.

The domain organisations of most short coronins (class-1, class-2, and unclassified coronins) are similar. They consist of the 390 amino-acid long coronin-domain followed by a short unique domain and a C-terminal short coiled-coil region (about 30-40 amino acids, Figure 3). The unique regions are conserved in branches (e.g. the vertebrates have similar regions, as do the arthropods, the nematodes, etc.), but are not conserved for major taxa (e.g. fungi, Metazoa, stramenopiles).

**Figure 4:**
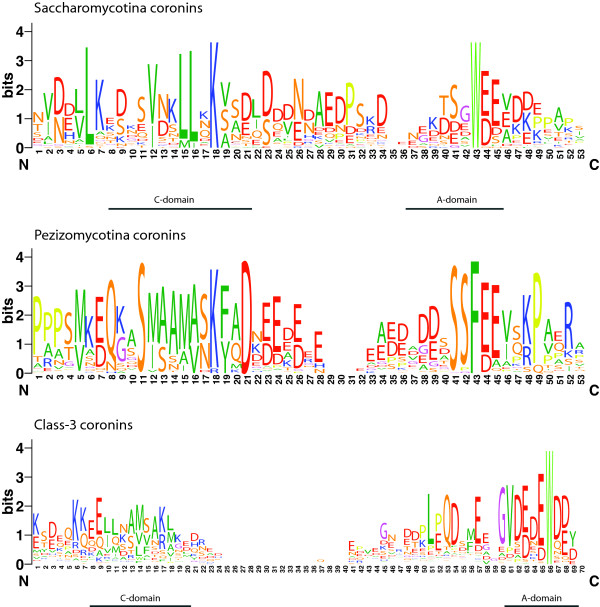
**Sequence conservation in the CA domains**. The sequence logos illustrate the sequence conservation within the multiple sequence alignments of the CA domains of the Saccharomycotina, the Pezizomycotina, and the class-3 coronins. The CA domains of the Saccharomycotina and the Pezizomycotina are located within the unique regions of the short coronins while the CA domain of the class-3 coronins is at the C-termini of the proteins like in WASP family proteins. The regions between the C and the A domains are of variable length.

The *Saccharomyces cerevisiae *coronin, ScCoro (CRN1), is known to bind to microtubules via its unique region between the β-barrel domain and the coiled-coil oligomerisation region (Figure 3, [30]). Two short regions showing homology to the microtubule-binding regions of MAP1B mediate this interaction. However, the MAP1B sequence motif is very short (about ten residues) and not very specific comprising mainly glutamate and lysine residues [30]. If the corresponding motifs in ScCoro are responsible for microtubule-binding then all yeast and *Schizosaccharomyces *coronins should be able to bind to microtubules because they contain motifs with similar amino acid compositions. A similar motif or region could not be identified in the Pezizomycotina coronins. While these supposed microtubule-binding regions mainly consist of glutamate, lysine, proline, serine, and threonine and are not even conserved in very closely related yeast species, the *Saccharomyces cerevisiae *coronin, *Sc*Coro, has very recently been described to contain a CA domain (C: central; A: acidic; [31]). This domain, with which *Sc*Coro activates and inhibits the ARP2/3 complex depending on concentration [31], is similar to CA domains in WASP family proteins [32]. The CA domain is well conserved but distinct within the Saccharomyceta clade (Pezizomycotina and Saccharomycotina, Figure 4).

Surprisingly, the coronins of the Tremellomycetes (e.g. *Filobasidiella/Cryptococcus *species) that belong to the Basidiomycota encode a C-terminal dUTPase domain (deoxyuridine triphosphatase domain) instead of the coiled-coil region (Figure 3). These coronin sequences are supported by many EST/cDNA clones for several of the *Filobasidiella *species extending from the coronin domain to the stop-codon. In addition to this dUTPase domain, the *Filobasidiella *species contain a further dUTPase in the genome that is conserved in the other Basidiomycotes, and also the other fungi. The dUTPase domains of the Tremellomycetes coronins contain all characteristic dUTPase domain motifs [33] and are therefore supposed to constitute enzymatically active domains. dUTPases typically form homotrimer active site architectures with all monomers contributing conserved residues to each of the three active sites [33]. Except for the prediction of trimerization of these coronins, which could be mediated by the dUTPase domains instead of the coiled-coil domains in the other coronins, it needs experimental data to link the function of actin filament structure remodelling by coronins to dUTP nucleotide hydrolysis in DNA repair by dUTPases.

### Class-3 coronins

Class-3 coronins (Type III coronins) comprise homologs that encode two coronin domains arranged in tandem [8]. These two coronin domains are separated by unique regions, and class-3 coronins do not encode coiled-coil domains. As recently reported [31] the class-3 coronins also encode a CA domain similar to the CA domain of the WASP family proteins at their C-termini (Figure 3). Based on the multiple sequence alignment of 112 class-3 coronins from all major branches of the eukaryotes the position of the C-region has slightly been adjusted in comparison with a previous analysis (Figure 4; [31]). Although the C-region of the class-3 coronins is not as conserved as similar regions in the yeast short coronins or in WASP family proteins, the characteristic pattern of hydrophobic residues concluded by a basic residue is visible in the homologs of all species (Figure 4). In contrast to the short coronins, the unique region between the C-terminal coronin-domain and the conserved CA-domain is short (20-30 amino acids).

Like for the short coronins the *Filobasidiella *species have surprising and species-specific tandem-coronins. The *Filobasidiella *class-3 coronins have a D-glycerate 3-kinase domain between the two coronin-domains (Figure 3). Only the termini of the *Filobasidiella *class-3 coronins are supported by EST/cDNA data, but long exons bridge the N-terminal coronin-domain and the glycerate 3-kinase domain, as well as the glycerate 3-kinase domain and the C-terminal coronin-domain. As found for the dUTPase domain of the short coronins, the *Filobasidiella *species contain an additional D-glycerate 3-kinase that has homologs in the other fungi and also in plants. Why it is advantageous to connect an actin-filament binding function to a glycerate 3-kinase needs experimental evaluation. The glycerate 3-kinase domain is not found in the class-3 coronins of the other Basidiomycotes. Except for the *Filobasidiella *species only the insects have long insertions between the two coronin-domains of their class-3 coronins. These insertions are highly conserved, about 300 residues long, and do not show any homology to known domains, sequence motifs, and other proteins.

In contrast to the related species *Rhizopus arrhizus *and *Phycomyces blakesleeanus *the coronin-3 of *Mucor circinelloides *consists of only the second coronin-domain of the tandem. We can exclude the possibility of this being an artefact of the genome assembly for three reasons. First, the genome sequence is continuous around *Muc*Coro3. Secondly, there is no homology to any part of the N-terminal coronin-domain of *Rha*Coro3 or *Phb*Coro3 in the genome although the sequence identity of the C-terminal coronin-domains is about 65%. And finally, there is a TATA-box shortly upstream of the *Muc*Coro3 gene. Because a coronin-3 has already been present in the most ancient eukaryote the loss of the N-terminal coronin-domain must be specific to *Mucor circinelloides*.

### Class-4 coronins

Based on the phylogenetic tree (Figure 1) and the domain composition of the protein homologs, another coronin class can be defined for which the *Dictyostelium discoideum *homolog, also called villidin [34], would be a representative (Figure 3). We suggest naming members of this class class-4 coronins. Most class-4 coronins consist of an N-terminal coronin-domain followed by three to four PH domains, four to five gelsolin domains, and a C-terminal villin headpeace domain (VHP). Class-4 coronins were identified in two of the major kingdoms of the eukaryotes, in excavates and opisthokonts. Furthermore, they are found in several of the sub-branches of the opisthokonts, in amoebae, fungi, and the fungi/metazoa incertae sedis branch. Because class-4 coronins from different species often contain different numbers of PH and gelsolin domains, domain gain and loss events must have happened in the respective branches or single species. However, there are not enough coronin-4 homologs identified yet to reconstruct the evolution of these regions. In addition to these multi-domain class-4 coronins there is a group of class-4 coronins that just consists of the conserved coronin domain and is restricted to some Amoebae species yet.

### Alternatively spliced coronins

**Figure 5:**
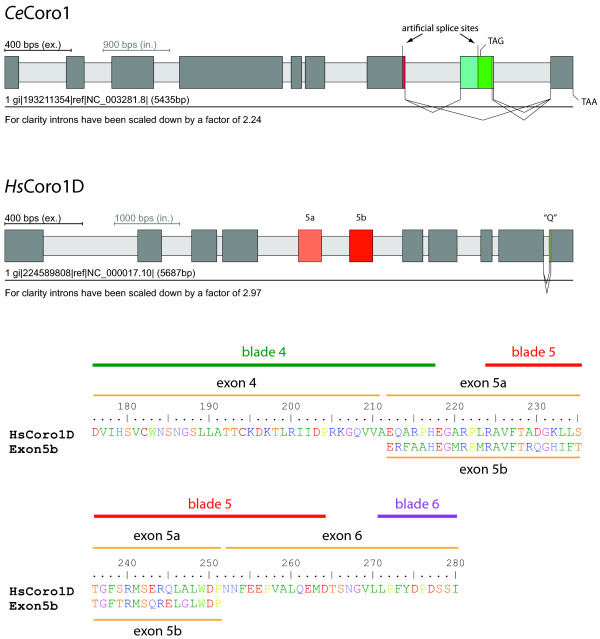
**Gene structures of alternatively spliced coronins**. The cartoons outline the gene structures of the alternatively spliced coronin-1 gene from *Caenorhabditis elegans, Ce*Coro1, and the coronin-1D gene from *Homo sapiens, Hs*Coro1D. The alternatively spliced *Ce*Coro1 gene contains a differentially included exon8, which has an additional alternative 3'-splice site, leading to three transcripts. The other two described splice sites, an alternative 3'-splice site of exon7 and an alternative 5'-splice site of exon8 [35], are most probably artificial. The *Hs*Coro1D gene contains a cluster of two mutually exclusive spliced exons, exon5a and exon5b, and an alternative 5'-splice site of exon10. Dark grey bars and light grey bars mark exons and introns, respectively, and alternative exons and splice sites are coloured.

Alternative splice forms have been reported for two coronin homologs: five variants of coronin from *Caenorhabditis elegans *[35], *Ce*Coro1 (Figure 5), and three variants for coronin-1C from human [36], *Hs*Coro1C. The described splice variants do not concern the beta-barrel domain but the structurally low-complexity region prior to the coiled-coil region in *Ce*Coro1 and elongations of the N-terminus of *Hs*Coro1C, respectively. In the reported analysis of *Ce*Coro1 [35] two splice sites (the alternative 3'-splice site of exon7 and the alternative 5'-splice site of exon8) do not obey the conventional splicing rules. Alternative 5'-splicing of exon8 would lead to a premature stop-codon. In the four additional *Caenorhabditis *strains analyzed here, *C. briggsae, C. japonica, C. remanei*, and *C.brenneri*, alternative 5'-splicing of exon8 would not lead to a premature stop-codon at the same position as in *C. elegans *but to transcripts of various lengths. The same accounts for several of the other available nematode coronin-1 genes. Given the high conservation of the nematode coronin-1 genes, especially the *Caenorhabditis *genes, and the completely uncommon nature of the potential splice sites, the reported alternative 3'-splice site of exon7 and 5'-splice site of exon8 are most probably artificial results. An alternative 3'-splice site has been reported for exon8 of *Ce*Coro1 comprising two amino acids [35]. Similar splice sites were identified in the genes of the other analyzed *Caenorhabditis *species but not in other nematodes. This splice site is thus also either an artificial result or specific for the *Caenorhabditis *branch. In addition, skipping of exon8 has also been reported to lead to an alternative transcript [35]. The intron position and reading frame of exon8 of *Ce*Coro1 is conserved in all analyzed nematode coronin-1's except for the *Strongyloides rattii *coronin-1, which consists of only one exon, and the *Pristionchus pacificus *coronin-1, which has introns at different positions. Compared to the full-length transcript, the other alternative splice forms of *Ce*Coro1 are of low abundance (see Figure two in [35]). Because the integrity of exon8 of *Ce*Coro1 (intron positions around the conserved coding sequence of exon8) is not conserved in nematodes but the corresponding amino-acid sequence, alternative splicing of nematode coronin-1 is either restricted to some sub-branches or an artificial result of the *Ce*Coro1 analysis.

Alternative splicing of human coronin-1C results in two additional transcripts derived from alternative transcription start sites encoded by an additional upstream exon, compared to the normal start site as found and conserved in all other coronin proteins [36]. These alternative splice forms seem to be restricted to modern primates (human, chimpanzee, gorilla, orangutan, and gibbon) and have been discussed in detail elsewhere [36].

We have identified alternative splice variants for coronin-1D (Figure 5), a coronin subfamily restricted to vertebrates. A cluster of two mutually exclusively spliced exons, exon5a and exon5b, was identified in all tetrapods. The amino-acid sequences corresponding to exon5 of the fish genes are more similar to exon5b than to exon5a. Thus, exon5a is the result of an exon duplication event that either occurred after the separation of tetrapods from fishes or at the onset of the vertebrates, where exon5a has been lost in the ancestor of the fishes. Exon5 represents the sequence of almost the entire fourth WD repeat (fifth blade in the β-propeller) starting in the middle of the fourth β-strand of blade four. By exchanging the fourth WD repeat the vertebrates could fine-tune the function of the coronin-1D beta-barrel domain. Vertebrate coronin-1D (CORO6) has not been analyzed experimentally yet and its specific function is unknown.

Further alternative transcripts are derived from mammalian coronin-1D genes by alternative 5'-splicing of the last exon, exon10. This alternative splicing results in one additional glutamine residue and is conserved in all 22 analyzed mammalian coronin-1D's except for *Ailuropoda melanoleuca *(giant panda), *Loxodonta africana *(elephant), *Myotis lucifugus *(little brown bat), and *Bos taurus *(cow).

### Oligomerization

Most of the short coronins have predicted coiled-coil domains at the C-terminus that are the bases for their supposed oligomerization. Initially, coronins have been proposed to form dimers [16], the most common form of coiled-coil multimerization. In the last decade, a few coronin homologs were biochemically purified and analyzed. Accordingly, the *Xenopus laevis *coronin-1C (XcoroninA) has been shown to form a dimer [37] while an oligomeric state has been found for human coronin-1C (coronin 3; [18], and the *Saccharomyces cerevisiae *coronin (CRN1) trimerizes [31]. Parallel trimer formation has also been shown in a crystal structure of the coiled-coil domain of mouse coronin-1A [20] revealing a conserved motif determining the trimeric structure: R_1_-[ILVM]_2_-X_3_-X_4_-[ILV]_5_-E_6_. In this motif arginine forms a salt-bridge with glutamate at the surface of the coiled-coil structure and the aliphatic side chain moieties of arginine and glutamate pack against the hydrophobic residues at positions 2 and 5 of the motif shielding them from solvent. Mutation of the arginine to lysine leads to a concentration-dependent equilibrium between trimers and tetramers with tetramers forming at high concentration, while mutation to alanine or norleucine leads to tetramers [20]. Mutation of the invariant arginine to glutamine in the trimerization motif of human matrilin-1 leads to tetramers [38]. Unfortunately, the switching of arginine and glutamate in the respective positions has not been analyzed yet. We would expect that such a switch should be as stable as the original motif. Thus, to predict the oligomerization state we have analyzed all coronin coiled-coil regions for the presence of the trimerization motif. Accordingly, all 233 class-1 coronins have the classical motif, except for *Dp*Coro1B and *Drp*Coro1B (*Drosophila pseudoobscura *and *persimilis*; Lys at position 1), and *Nv*Coro1 (*Nematostella*; Cys at position 2), and are thus predicted to form trimers. This would include the *Xenopus *Coro1C that has, however, been shown to exist as a dimer [37]. The situation is more diverse for the class-2 coronins. The invertebrate coronins contain the trimerization motif, except for *Amq*Coro2 (*Amphimedon*; Ser at P1), *Her*Coro2A (*Helobdella*; Lys at P1), *Her*Coro2B (Phe at P1), *Myd*Coro2 (*Mayetiola*; Gln at P6), and the nematode class-2 coronins (Cys at P2). Almost all fish class-2 coronins contain the trimerization motif, but the other vertebrate class-2 coronins have conserved mutations. The tetrapod class-2A coronins encode a glutamine instead of the invariant arginine, which would turn them to tetramers in analogy to matrilin-1 [38]. The tetrapod class-2B coronins contain glutamine instead of the glutamate at position 6 of the motif, a substitution whose effect has not been analyzed yet.

About half of the analyzed fungal coronins have the classical trimerization motif. The most common substitutions that are found in all Schizosaccharomyces and most Basidiomyota coronins are lysines or glutamines instead of the arginine at position 1. While the coiled-coil region is conserved in general, substitutions happened in specific species but not in whole branches (except for the Schizosaccharomyces). Therefore, we would expect all fungal coronins to form trimers. All Amoeba coronins, the Stramenopiles coronins (exceptions: *Frc*Coro a His at P1, *Bh*Coro_B a Asn at P6, *Aua*Coro_B a Lys at P1), the *Trichomonas *and *Naegleria *coronins contain the classical trimerization sequence motif in the coiled-coil region. Interestingly, the kinetoplastid coronins have the salt-bridge switched in the motif and should thus also be able to form trimers. From the Alveolata, only the Ciliophora (e.g. *Tetrahymena*) and Coccidia (e.g. *Toxoplasma gondii*) coronins contain coiled-coil domains, and only the Coccidia contain the trimerization motif.

These are, however, predictions based on the existence of the proposed trimerization motif. The motif has been identified in 86% of all short, autonomous, and parallel three-stranded coiled-coils while it is also observed in 9% of the antiparallel trimers and in 5% of the parallel and antiparallel dimers [20]. Thus, although most short coronins are predicted to form trimers some might nevertheless function in other oligomeric states in the cell. The oligomerization state can ultimately only be shown in experiments, which have, however, been done for just a few of the coronins yet.

### F-actin binding

**Figure 6:**
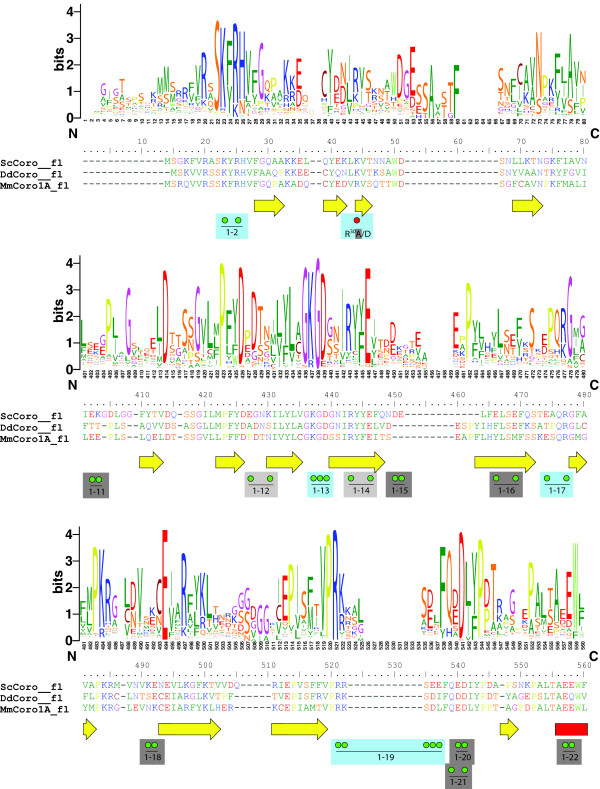
**Sequence conservation within the actin binding region**. The sequence logos illustrate the sequence conservation within the multiple sequence alignments of the coronin domains. Here, only the N- and C-termini of the coronin domains are shown because most of the residues implicated in actin binding map to these regions. For the representation of the entire coronin domain see Additional file 5. For better orientation, the sequences of three representative coronins are shown: the yeast coronin as the main target of mutagenesis experiments, the *Dictyostelium *coronin as the founding member of the protein family, and the murine coronin-1A of which the crystal structure is known. Secondary structural elements as determined from the crystal structure are drawn as yellow arrows (β-strands) and red boxes (α-helices). Green dots point to amino acids of *Sc*Coro that have been mutated to alanine [41] and red dots highlight mutagenesis studies in *Hs*Coro1B [40]. Light-blue boxes highlight mutations that abolished actin binding, dark-grey boxes represent mutations that did not influence actin binding, and light-grey boxes point to mutations in yeast coronins that could not be expressed and tested.

F-actin binding is one of the common properties of coronin proteins. The extended multiple sequence alignment presented here together with the recently determined crystal structure of murine coronin-1A [17] now allows a reevaluation of previous mutagenesis studies. Truncation studies have shown that the coronin domain, including the β-propeller and its C-terminal extension, is necessary for F-actin binding [30,39]. Mapping the sequence conservation within 13 short coronin members onto the surface of the crystal structure revealed two regions, one formed by blades 1, 6, and 7 and one formed by blades 6 and 7 and a portion of the C-terminal extension, to represent possible actin binding sites [17]. Subsequently, several surface-exposed charged amino acids have been mutated to alanine or substituted by reversed charges in human coronin-1B and their F-actin binding affinity has been analyzed ([40], Figure 6 red dots, see also Additional file 5). Only the R30D mutation abolished actin binding in vitro. Although an arginine is the most prevalent amino acid at this position it is often substituted by a lysine or a proline (Figure 6). The multiple sequence alignment of the coronins also does not show a trend towards a class-specific substitution. For example, while a proline is found at this position in all vertebrate class-2 coronins, arginines, lysines, asparagines, prolines, threonins, and tyrosins are found in invertebrate class-2 coronins. At least negatively charged amino acids are not found in any of the coronin domains at this position. Recently, systematic mutagenesis of charged surface-exposed residues of yeast coronin revealed a patch of residues extending over the top and one side of the β-propeller that abolished actin binding when mutated to alanine (Figure 6, green dots [41]). The analysis of the conservation within the coronin proteins shows that many of the substitutions in both studies have been performed on marginally conserved residues (e.g. E^215^A/K, K^216^A/E, 1-11, 1-15, 1-16). Thus, it is not surprising that coronins with mutations of these residues are able to bind F-actin. As actin binding is one of the common functions of coronins and actins belong to the highest conserved protein families the actin binding surface of the coronins is also expected to be highly conserved. Most of the residues that were found to abolish actin binding when mutated to alanine are strongly conserved (Figure 6). The few residues that are highly conserved but do not influence actin binding might be interaction sites for other proteins like cofilin.

## Discussion

Here, we have analyzed 723 coronins from 358 species. For 323 species whole genome sequence data was available allowing a "holistic" analysis of the coronin protein family. In addition, the whole genome assemblies of 69 species have been analyzed that in the end did not contain any coronin homolog. These species include Rhodophyta (*Cyanidioschyzon, Galdieria*), Viridiplantae, Microsporidia, Formicata (*Giardia*), and Haptophyceae (*Emiliania*). A sequence alignment of the coronin proteins was created and extensively improved manually. The phylogenetic analysis of the conserved coronin domain, which is also included in the crystal structure [17], using the Bayesian method showed that the grouping of the coronins is completely in accordance with the latest phylogeny of the eukaryotic species (Figure 1, [27-29]). Subsequently, we analyzed the coronin tree with respect to established and proposed classifications defining subfamilies. Two major schemes are currently in use, the old one established by the HGNC [8] and a more recent one expanding the number of classes from three to twelve [22]. Essentially, the later classification re-defines subclasses of the HGNC scheme as separate classes, e.g. 1A and 1B become class-4 and class-1, respectively, and groups some branches to new classes. However, some coronins still remained unclassified and several classes have been proposed, like the invertebrate metazoan classes 8 and 9, although the contributing members did not form monophyletic branches in the underlying protein family tree. The proposed classes 10 and 12 contain members of unrelated taxonomic branches, probably because these coronins were adjacent in the tree figure. In addition, the Entamoeba tandem-coronin did not group to the other tandem-coronins. Thus, this classification is not consistent with the taxonomy of the eukaryotes. In addition, homologs of major branches were missing in the analysis like those from stramenopiles. We do not intend to add confusion to the classification of the coronin family but want to suggest a reliable and, considering future genome sequencing projects, expandable scheme. Two major reasons support the future use of the HGNC scheme although it needs some minor adjustments. The classification by Morgan and Fernandez [22] of coronins outside the metazoans is not consistent with the latest taxonomy of the eukaryotes and therefore not adaptable to our more comprehensive coronin tree. In addition, it is well known that two whole-genome-duplications are the reason for the expansion of gene homologs at the origin of the vertebrates [42], while another whole-genome-duplication happened at the origin of the Actinopterygii [43,44]. Thus retaining the orthology between non-vertebrate and vertebrate coronins in class-numbers would be desirable but has also been abandoned by Morgan and Fernandez [22]. Here, we adapted the HGNC classification except for renaming CORO6 and CORO7 (HGNC) to coronin-1D and coronin-3, respectively, numbering additional fish coronins as coronin-1E, coronin-2C, and coronin-2D, and defining the new coronin class-4. The term "class" is equivalent to the term "Type" used by the Bear group in recent reviews [8,45]. However, we prefer the term "class" to be consistent with the terminology used for other protein families (e.g. the myosin family [46,47]) and therefore to facilitate the work with databases and search engines in the future.

Class-4 coronins represent a new type of coronins that are present in Excavata (*Naegleria gruberi*), Amoebae, fungi (*Spizellomyces punctatus*), and the Fungi/Metazoa incertae sedis branch. Most class-4 coronins consist of the N-terminal coronin domain followed by two to three PH, four to five gelsolin, and a C-terminal VHP domain. The first representative of this subfamily has been identified in *Dictyostelium discoideum *and called villidin because of the homology of its gelsolin and VHP domains to villin [34]. The homology of villidins WD-repeat region to coronin has been recognized later on [48,49] suggesting villidins origin through a fusion of the coronin domain with villin. Villin is the founding member of a superfamily of proteins containing three to six gelsolin domains (reviewed in [48,50]). Like villidin (class-4 coronins), villin, supervillin, and protovillin also contain a C-terminal VHP domain. Alignment of villin to the class-4 coronins gelsolin domains shows that the class-4 coronins have lost the first gelsolin domain of villin. The first gelsolin domain of villin is associated with dimerization, actin filament capping, nucleation, and bundling, and G-actin binding [50]. Thus, class-4 coronins do not play a role in these activities via their gelsolin domains [34]. However, villin contains three phospholipid-binding domains, two preceding the second gelsolin domain and one overlapping with the VHP domain. These phospholipid-binding domains are conserved in class-4 coronins and are most probably responsible for their association with internal membranes like Golgi-structures and ER-membranes [34].

**Figure 7:**
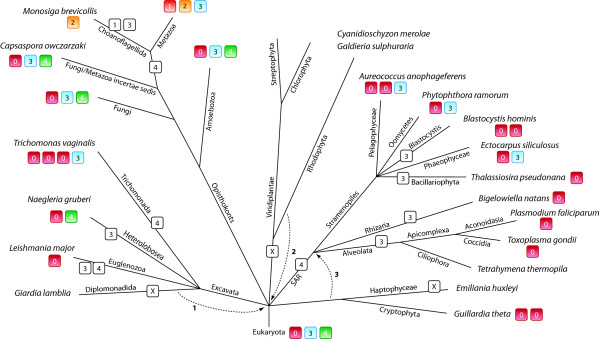
**Evolution of the coronin protein family with respect to the species evolution**. Schematic representation of the most widely accepted eukaryotic tree of life. Branch lengths are arbitrary. The coronin inventories of certain taxa and specific species have been plotted to the tree with class numbers given in colour-coded boxes. "O" stands for "Orphan", the unclassified short coronins. The numbers on the arrows refer to alternative placing of the respective taxa: **1**: The independence of the Diplomonadida (instead of grouping them to the superkingdom Excavata) is supported by [51]. **2**: The monophyly of the Rhodophyta is supported by [28,55]. **3**: Grouping the Haptophyceae and Cryptophyta to the SAR is supported by [55-57].

**Figure 8:**
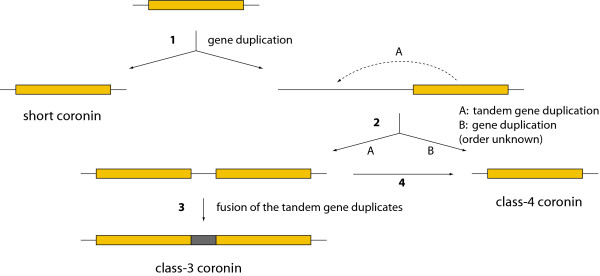
**Evolution of the coronin classes**. The cartoon shows the different gene duplication and fusion events that led to the formation of the short coronins, the class-3 coronins, and the class-4 coronins.

To reveal the evolution of the coronin family and to determine the coronin repertoire of the last common ancestor of the eukaryotes, we plotted the coronin inventory of several representative species, whose genome sequences are available and whose coronin inventories are therefore complete, on the most widely agreed tree of the eukaryotes (Figure 7). However, especially the grouping of taxa that emerged close to the origin of the eukaryotes remains highly debated. Therefore, alternative branchings are also indicated in the tree. The phylogeny of the supposed supergroup Excavata is the least understood because only a few species of this branch have been completely sequenced so far. While the grouping of the Heterolobosea, Trichomonada, and Euglenozoa into the Excavata is found in most analyses, the grouping of the Diplomonadida as separate phylum or as part of the Excavata is still debated (arrow 1 [51]). Also, some analyses group the red algae of the Rhodophyta branch to the Viridiplantae [52-54] and others support their independence (arrow 2; [28,55]). According to most of the recent phylogenetic analyses, the Alveolata, Rhizaria, and Stramenopiles form the superfamily SAR [27,54]. The placement of the Haptophyceae and Cryptophyta to the SAR is still highly debated. Although several analyses are in favour to this grouping (arrow 3; [55-57]) most analyses are in contrast [27-29,53,54]. Short coronins containing the N-terminal coronin domain and the C-terminal oligomerization domain have been found in all branches except Diplomonadida, Haptophyceae, and Viridiplantae/Rhodophyta. The phylogenetic grouping of the species based on the phylogenetic tree of the coronin domains showed that the coronins with different domain compositions (containing dUTPase domains, ARP2/3 binding domains, no coiled-coil regions) are species-specific developments based on domain loss and gain events while the corresponding species correctly group together inside the respective branches. Class-3 coronins are also found in all major eukaryotic superkingdoms that contain coronins. We did not identify any species that contains exclusively a class-3 coronin suggesting that encoding a class-3 coronin is a plus for the species but not a necessity. Class-4 coronins were found in two of the four coronin-containing superkingdoms, the Excavata and the Opisthokonts. Several major sub-branches of the Opisthokonts contain class-4 coronins, the Amoebozoa, the Fungi, and the Fungi/Metazoa incertae sedis branch. However, the evolution of the class-4 coronins rather seems to be determined by gene-loss events. This distribution of the coronin classes demonstrates that the last common ancestor of the eukaryotes must have contained a short coronin as well as a tandem coronin (class-3), and most probably even a class-4 coronin. In the coronin-family tree (Figure 1) the C-terminal coronin-domains of the class-3 coronins group closer to the short coronins than the N-terminal coronin-domains. This suggests a three-step invention of the class-3 coronin (Figure 8): First a gene duplication of the short coronin happened (**1**). The new copy was subsequently copied twice but the order of these events could not be determined (**2**). One copy has been distributed in a different genomic region resulting in the class-4 coronin after fusion to a copy of the villin gene (**2B**). The other copy resulted in a tandem gene duplicate in which the new copy was placed at the 5' site of the original gene (**2A**). The tandem gene duplicate subsequently fused to build the class-3 coronin prototype (**3**). It could also be possible that the coronin domain copy, which led to the class-4 coronin, would have been produced as a copy of the 3' coronin of the then already existing tandem gene duplicate (**4**).

At the origin of the Metazoa and Choanoflagellida branches another gene duplication event led to two distinct classes, class-1 coronins and class-2 coronins (Figure 7). The further evolution of the short coronins in the invertebrate branches is determined by species-specific gene-loss and gene-duplication events (Figure 2). This view is, however, based on the species whose genomes are available today and might change as soon as sequencing of more related species reveals subtypes of the class-1 and class-2 coronins in major invertebrate branches. At the origin of the vertebrates the two well-known whole-genome duplications (2R, [42]) resulted in several subtypes of both the class-1 and class-2 coronins. The subsequent third whole genome duplication in the fish-lineage [43,44] led to even more gene duplicates. Subsequent to this boost of coronin homologs at the onset of the vertebrates branch-specific gene deletions happened, like the loss of the class-1B variants in fishes and the class-1A loss in birds (Figure 2).

The short coiled-coil region including the trimerization motif R-[VILM]-X-X-[VIL]-E is an accomplishment of the most ancient short coronin because it is found in coronins of all branches of the eukaryotic tree. It has been retained without major mutations for a long evolutionary time. This is exemplified by the fact that changes, which might lead to other oligomerization states, are species-specific or have been introduced in very recently separated branches.

## Conclusions

The phylogenetic tree based on the coronin domains of 723 homologs from 358 species allowed grouping the coronin proteins into four classes: Class-1 (Type I) and class-2 (Type II) comprise short coronins and resulted from a gene duplication of a short coronin at the onset of the halozoans. Short coronins are characterized by an N-terminal coronin domain followed by a unique domain and a C-terminal short coiled-coil region. The coiled-coil domain of almost all short coronins contains a trimerization motif that must therefore have already existed in the last common ancestor of the eukaryotes. Class-3 (Type III) coronins comprise coronins with two coronin-domains arranged in tandem and have been found in species of all eukaryotic kingdoms that contain coronins. Class-4 (Type IV) coronins encode fusions of the coronin domain to villin and have been identified in Excavata and Opisthokonts although most of these species subsequently lost the class-4 homolog. Hence, the last common ancestor of the eukaryotes must have contained a short coronin and a class-3 coronin, and most probably a class-4 coronin.

## Methods

### Identification and annotation of the coronin family proteins

The coronin genes have been identified by TBLASTN searches against the sequenced eukaryotic genomes, which have been obtained via lists available from the diArk database [23,58]. All hits were manually analyzed at the genomic DNA level. Datasets of predicted proteins produced by the sequencing consortia often miss homologs, and predicted proteins contain mispredicted exons and introns in many cases, necessitating manual assembly and annotation. The correct coding sequences were identified with the help of the multiple sequence alignments of all coronin proteins. As the amount of protein sequences increased (especially the number of sequences in taxa with few representatives), many of the initially predicted sequences were reanalyzed to correctly identify all exon borders. Where possible, EST data available from the NCBI EST database has been analyzed to help in the annotation process. In addition, coronin homologs from cDNA projects or single-gene analyses have been obtained by TBLASTN searches against the NCBI nr database [59]. Gene structures have been reconstructed using WebScipio [25] as far as genomic sequence data was available. All sequence related data (names, corresponding species, GenBank ID's, alternative names, corresponding publications, domain predictions, gene structure reconstructions, and sequences) and references to genome sequencing centres are available through CyMoBase [60,61].

### Generating the multiple sequence alignment

The multiple sequence alignment of the coronin family has been built and extended during the process of annotating and assembling new sequences. The initial alignment has been generated from the first about 50 non-validated sequences obtained from NCBI using the ClustalW software with standard settings [62]. During the following correction of the sequences (removing wrongly annotated sequences and filling gaps) the alignment has been adjusted manually. Subsequently, every newly predicted sequence has been preliminary aligned to its supposed closest relative using ClustalW, the aligned sequence added to the multiple sequence alignment of the coronins, and the coronin alignment adjusted manually during the subsequent sequence validation process. We have also retained the integrity of the primary sequence within the secondary structural elements that have been determined from the crystal structure (e.g. sequence gaps have only been introduced in known loop regions). Still, many gaps in sequences derived from low-coverage genomes remained. In those cases, the integrity of the exons surrounding the gaps has been maintained (gaps in the genomic sequence are reflected as gaps in the multiple sequence alignment). The unique and coiled-coil regions are completely divergent in sequence and length and were therefore aligned manually. The domain compositions of the short coronin, the class-3, and the class-4 coronins are different and regions outside the N-terminal coronin domain were only aligned within these groups. The C-terminal coronin domains of the class-3 coronins were separately included in the multiple sequence alignment of the coronins, in addition to being aligned as part of their class.

### Building trees

For calculating phylogenetic trees only full-length and partial sequences were included in the alignment. The phylogenetic trees were generated based on the conserved coronin domains (corresponding to amino acids 1-386 of *Hs*Coro1A) using two different methods: 1. Maximum likelihood (ML) using the LG model with estimated proportion of invariable sites and bootstrapping (1,000 replicates) using RAxML [63]. 2. Posterior probabilities were generated using MrBayes v3.1.2 [64] with the MPI option [65]. Two independent runs with 15,000,000 generations, four chains, and a random starting tree were computed using the mixed amino-acid option. From the 32,000th generation MrBayes used the Wag model [66]. Using ProtTest [67], the LG model [68], which is, however, not implemented in MrBayes, was determined to provide a slightly better fit to the data than the Wag model. Trees were sampled every 1,000th generation and the first 25% of the trees were discarded as "burn-in" before generating a consensus tree.

### Domain and motif prediction

Protein domains were predicted using the SMART [69] and Pfam [70] web server. The leucine zipper motifs have been identified using the Prosite database [71]. The CA domains have been identified by visual inspection of the manual sequence alignment of the coronins and motif comparisons with CA domains of WASP family proteins available at CyMoBase (unpublished data, [61]). Graphical representations of the sequence patterns have been generated with WebLogo [72].

## Authors' contributions

CE and MK assembled coronin sequences, performed data analysis and wrote the manuscript. BH performed the phylogenetic analysis. All authors read and approved the final manuscript.

## Supplementary Material

Additional file 1 **Sequence alignment of the coronins **The file contains the alignment of the full-length sequences of the coronins in fasta-format. The data can also be downloaded from CyMoBase [61]. Click here for file

Additional file 2 **MrBayes tree of the coronin family **This file contains the phylogenetic tree calculated with MrBayes including posterior probability values that has been the basis for Figure 1. Here, the tree is plotted in an extended way so that every coronin can be found and compared easily. Click here for file

Additional file 3 **RAxML tree of the coronin family **This file contains the phylogenetic tree calculated with RAxML including bootstrap values. The tree is plotted in an extended way so that every coronin can be found and compared easily. Click here for file

Additional file 4 **Coronin repertoire of all eukaryotes analyzed **Complete table of the coronin inventories of 358 eukaryotes. Click here for file

Additional file 5 **Conserved residues in the coronin domain **This figure contains the sequence conservation of the entire coronin domain including all mutagenesis experiments as described in Cai *et al*. [40] and Gandhi *et al*. [41]. Click here for file
