# Supplementary material for: A holistic phylogeny of the coronin gene family reveals an ancient origin of the tandem-coronin, defines a new subfamily, and predicts protein function
Source: BMC Evol Biol. 2011 Sep 25;11:268. doi: 10.1186/1471-2148-11-268 (PMC3203266; doi:10.1186/1471-2148-11-268)
Supplement: Additional file 2 — MrBayes tree of the coronin family This file contains the phylogenetic tree calculated with MrBayes including posterior probability values that has been the basis for Figure 1. Here, the tree is plotted in an extended way so that every coronin can be found and compared easily. [file 1471-2148-11-268-S2.PDF]

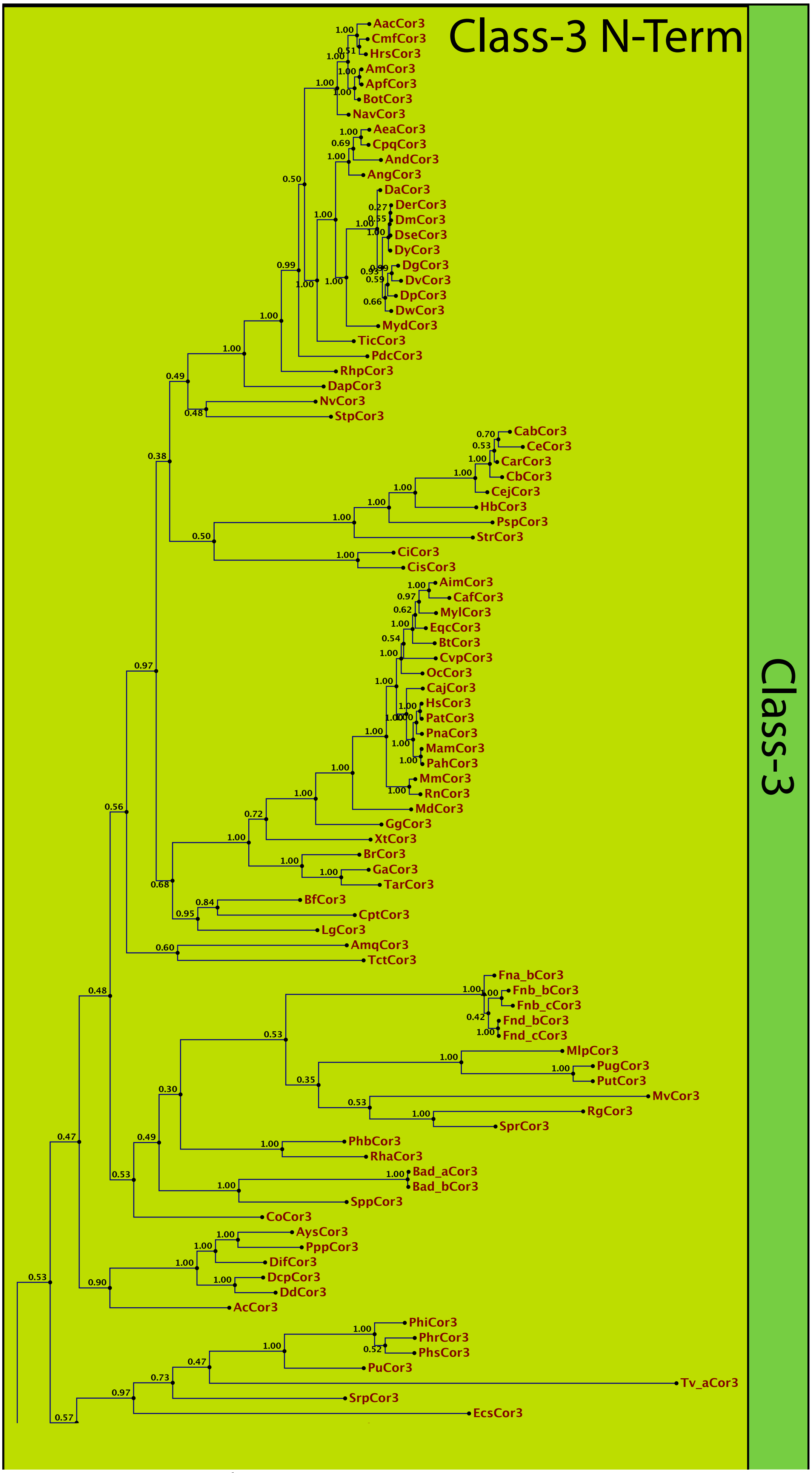
Class-3 N-Term
Class-3
AacCor3
CmfCor3
HrsCor3
AmCor3
ApfCor3
BotCor3
NavCor3
AeaCor3
CpqCor3
AndCor3
AngCor3
DaCor3
DerCor3
DmCor3
DseCor3
DyCor3
DgCor3
DvCor3
DpCor3
DwCor3
MydCor3
TicCor3
PdcCor3
RhpCor3
DapCor3
NvCor3
StpCor3
CabCor3
CeCor3
CarCor3
CbCor3
CejCor3
HbCor3
PspCor3
StrCor3
CiCor3
CisCor3
AimCor3
CafCor3
MylCor3
EqcCor3
BtCor3
CvpCor3
OcCor3
CajCor3
HsCor3
PatCor3
PnaCor3
MamCor3
PahCor3
MmCor3
RnCor3
MdCor3
GgCor3
XtCor3
BrCor3
GaCor3
TarCor3
BfCor3
CptCor3
LgCor3
AmqCor3
TctCor3
Fna_bCor3
Fnb_bCor3
Fnb_cCor3
Fnd_bCor3
Fnd_cCor3
MlpCor3
PugCor3
PutCor3
MvCor3
RgCor3
SprCor3
PhbCor3
RhaCor3
Bad_aCor3
Bad_bCor3
SppCor3
CoCor3
AysCor3
PppCor3
DifCor3
DcpCor3
DdCor3
AcCor3
PhiCor3
PhrCor3
PhsCor3
PuCor3
Tv_aCor3
SrpCor3
EcsCor3

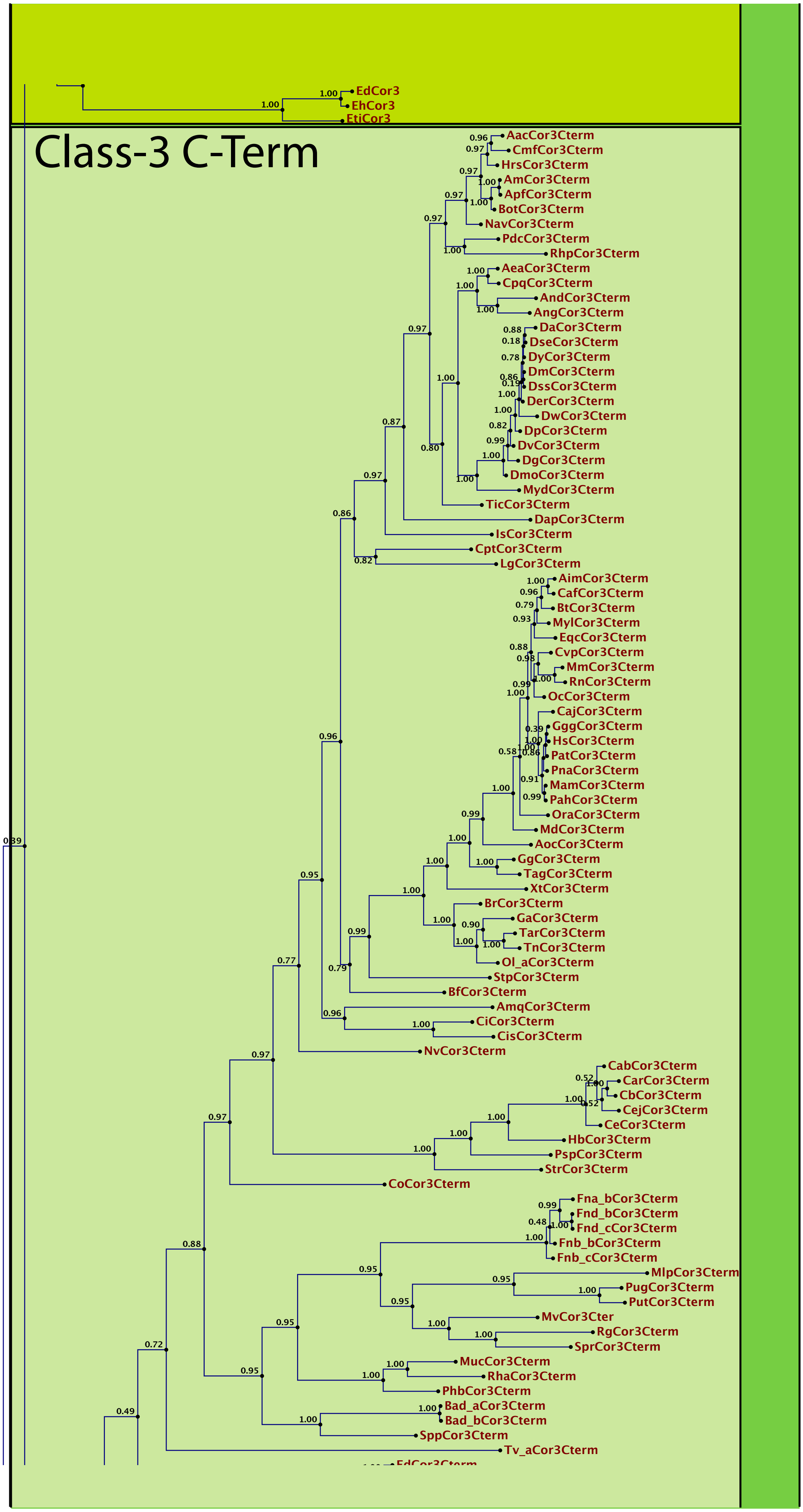
EdCor3
EhCor3
EtiCor3
Class-3 C-Term
AacCor3Cterm
CmfCor3Cterm
HrsCor3Cterm
AmCor3Cterm
ApfCor3Cterm
BotCor3Cterm
NavCor3Cterm
PdcCor3Cterm
RhpCor3Cterm
AeaCor3Cterm
CpqCor3Cterm
AndCor3Cterm
AngCor3Cterm
DaCor3Cterm
DseCor3Cterm
DyCor3Cterm
DmCor3Cterm
DssCor3Cterm
DerCor3Cterm
DwCor3Cterm
DpCor3Cterm
DvCor3Cterm
DgCor3Cterm
DmoCor3Cterm
MydCor3Cterm
TicCor3Cterm
DapCor3Cterm
IsCor3Cterm
CptCor3Cterm
LgCor3Cterm
AimCor3Cterm
CafCor3Cterm
BtCor3Cterm
MylCor3Cterm
EqcCor3Cterm
CvpCor3Cterm
MmCor3Cterm
RnCor3Cterm
OcCor3Cterm
CajCor3Cterm
GggCor3Cterm
HsCor3Cterm
PatCor3Cterm
PnaCor3Cterm
MamCor3Cterm
PahCor3Cterm
OraCor3Cterm
MdCor3Cterm
AocCor3Cterm
GgCor3Cterm
TagCor3Cterm
XtCor3Cterm
BrCor3Cterm
GaCor3Cterm
TarCor3Cterm
TnCor3Cterm
Ol_aCor3Cterm
StpCor3Cterm
BfCor3Cterm
AmqCor3Cterm
CiCor3Cterm
CisCor3Cterm
NvCor3Cterm
CabCor3Cterm
CarCor3Cterm
CbCor3Cterm
CejCor3Cterm
CeCor3Cterm
HbCor3Cterm
PspCor3Cterm
StrCor3Cterm
CoCor3Cterm
Fna_bCor3Cterm
Fnd_bCor3Cterm
Fnd_cCor3Cterm
Fnb_bCor3Cterm
Fnb_cCor3Cterm
MlpCor3Cterm
PugCor3Cterm
PutCor3Cterm
MvCor3Cter
RgCor3Cterm
SprCor3Cterm
MucCor3Cterm
RhaCor3Cterm
PhbCor3Cterm
Bad_aCor3Cterm
Bad_bCor3Cterm
SppCor3Cterm
Tv_aCor3Cterm

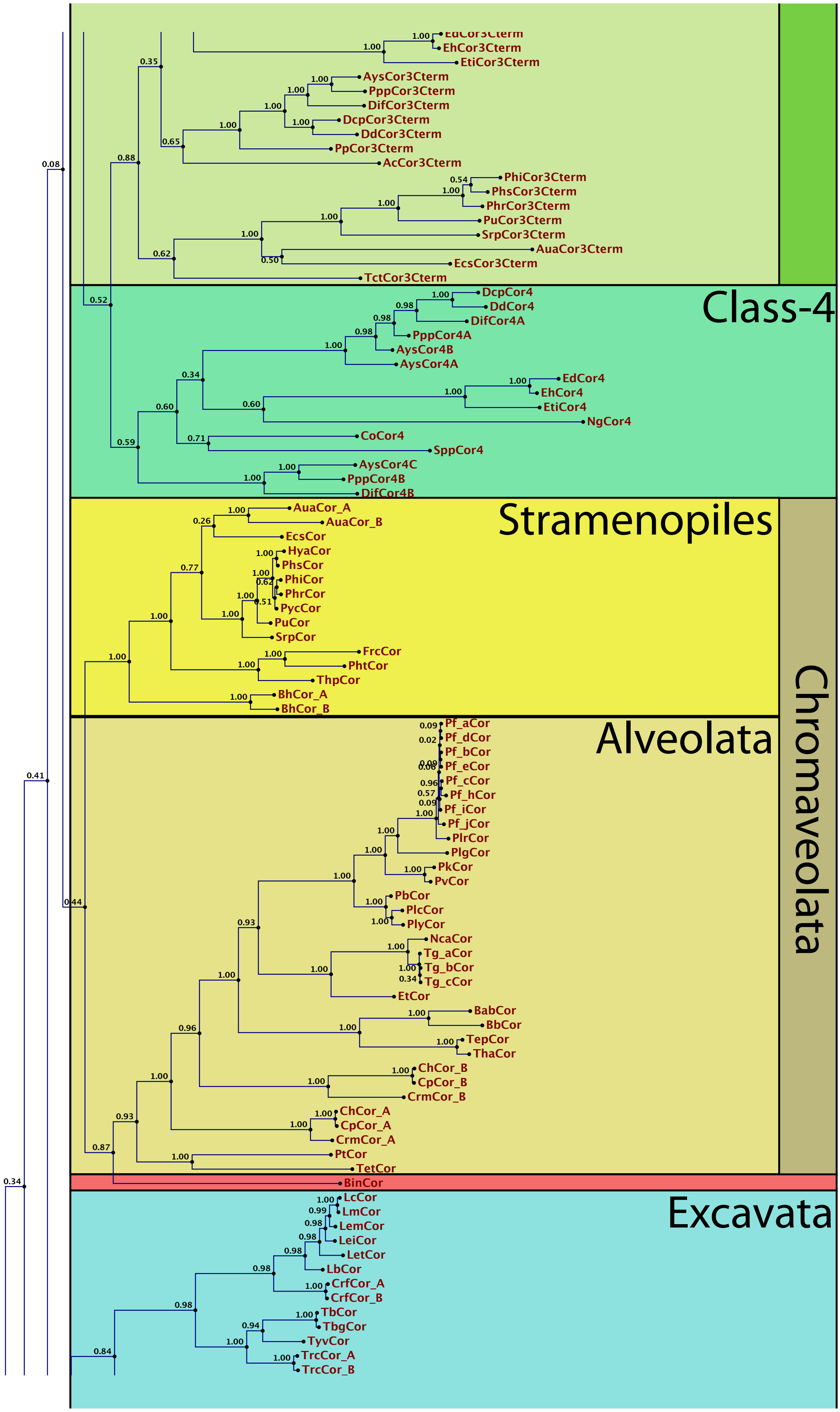
Class-4
Stramenopiles
Alveolata
Chromaveolata
Excavata
EhCor3Cterm
EtiCor3Cterm
AysCor3Cterm
PppCor3Cterm
DifCor3Cterm
DcpCor3Cterm
DdCor3Cterm
PpCor3Cterm
AcCor3Cterm
PhiCor3Cterm
PhsCor3Cterm
PhrCor3Cterm
PuCor3Cterm
SrpCor3Cterm
AuaCor3Cterm
EcsCor3Cterm
TctCor3Cterm
DcpCor4
DdCor4
DifCor4A
PppCor4A
AysCor4B
AysCor4A
EdCor4
EhCor4
EtiCor4
NgCor4
CoCor4
SppCor4
AysCor4C
PppCor4B
DifCor4B
AuaCor_A
AuaCor_B
EcsCor
HyaCor
PhsCor
PhiCor
PhrCor
PycCor
PuCor
SrpCor
FrcCor
PhtCor
ThpCor
BhCor_A
BhCor_B
Pf_aCor
Pf_dCor
Pf_bCor
Pf_eCor
Pf_cCor
Pf_hCor
Pf_iCor
Pf_jCor
PlrCor
PlgCor
PkCor
PvCor
PbCor
PlcCor
PlyCor
NcaCor
Tg_aCor
Tg_bCor
Tg_cCor
EtCor
BabCor
BbCor
TepCor
ThaCor
ChCor_B
CpCor_B
CrmCor_B
ChCor_A
CpCor_A
CrmCor_A
PtCor
TetCor
BinCor
LcCor
LmCor
LemCor
LeiCor
LetCor
LbCor
CrfCor_A
CrfCor_B
TbCor
TbgCor
TyvCor
TrcCor_A
TrcCor_B

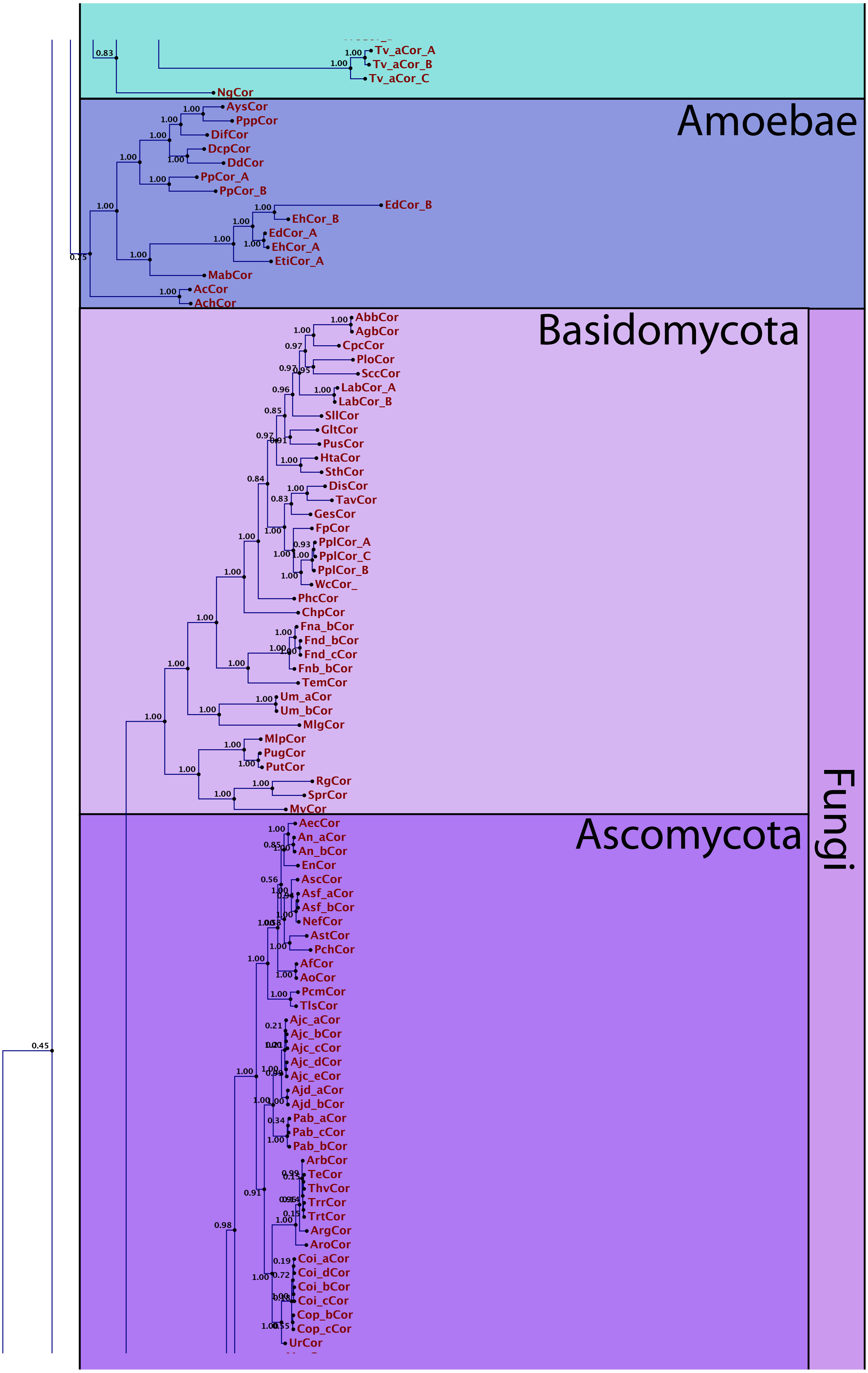
Tv_aCor_A
Tv_aCor_B
Tv_aCor_C
NgCor
Amoebae
AysCor
PppCor
DifCor
DcpCor
DdCor
PpCor_A
PpCor_B
EdCor_B
EhCor_B
EdCor_A
EhCor_A
EtiCor_A
MabCor
AcCor
AchCor
Basidomycota
AbbCor
AgbCor
CpcCor
PloCor
SccCor
LabCor_A
LabCor_B
SllCor
GltCor
PusCor
HtaCor
SthCor
DisCor
TavCor
GesCor
FpCor
PplCor_A
PplCor_C
PplCor_B
WcCor_
PhcCor
ChpCor
Fna_bCor
Fnd_bCor
Fnd_cCor
Fnb_bCor
TemCor
Um_aCor
Um_bCor
MlgCor
MlpCor
PugCor
PutCor
RgCor
SprCor
Ascomycota
AecCor
An_aCor
An_bCor
EnCor
AscCor
Asf_aCor
Asf_bCor
NefCor
AstCor
PchCor
AfCor
AoCor
PcmCor
TlsCor
Ajc_aCor
Ajc_bCor
Ajc_cCor
Ajc_dCor
Ajc_eCor
Ajd_aCor
Ajd_bCor
Pab_aCor
Pab_cCor
Pab_bCor
ArbCor
TeCor
ThvCor
TrrCor
TrtCor
ArgCor
AroCor
Coi_aCor
Coi_dCor
Coi_bCor
Coi_cCor
Cop_bCor
Cop_cCor
UrCor
Fungi

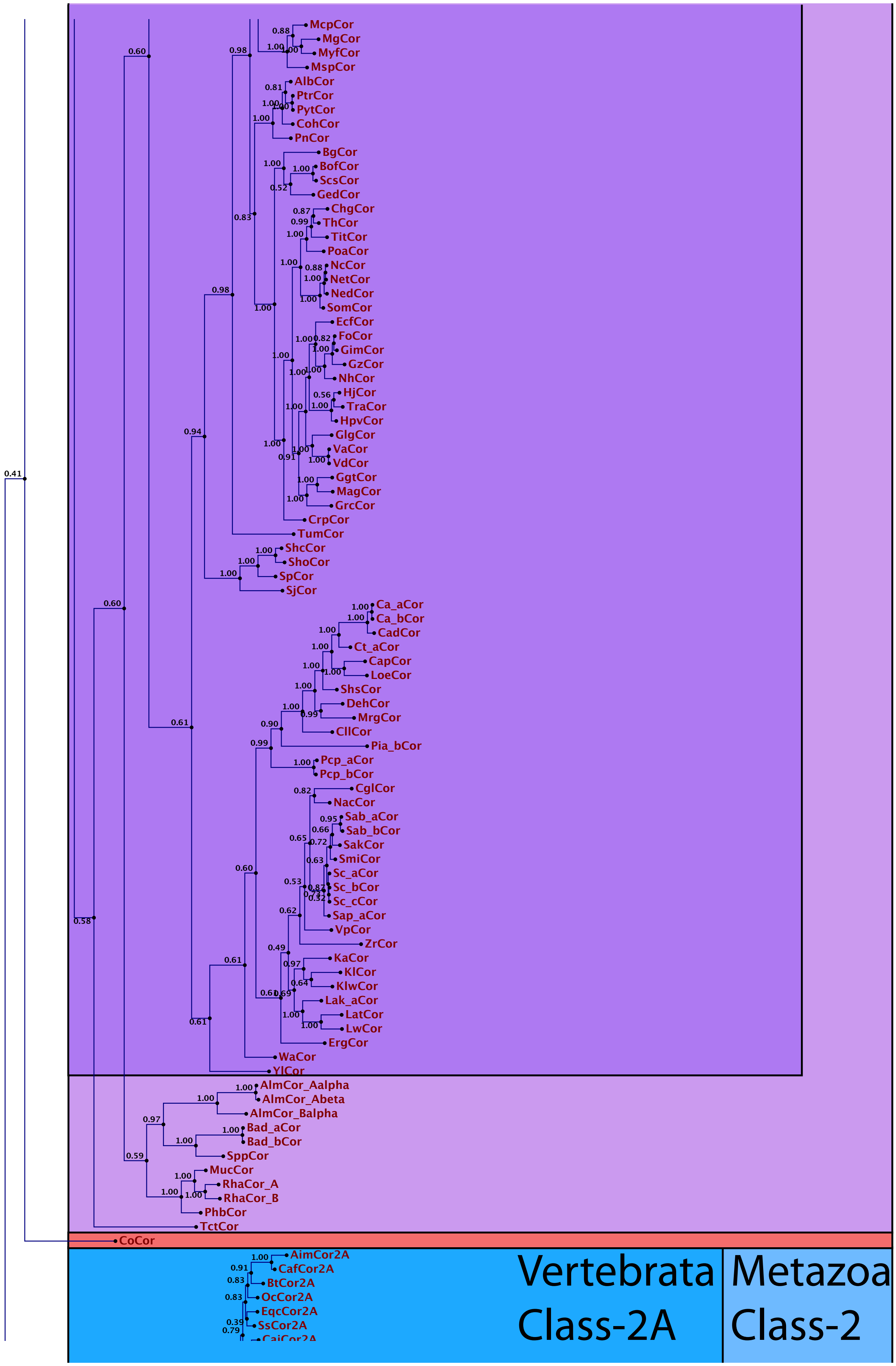
McpCor
MgCor
MyfCor
MspCor
AlbCor
PtrCor
PytCor
CohCor
PnCor
BgCor
BofCor
ScsCor
GedCor
ChgCor
ThCor
TitCor
PoaCor
NcCor
NetCor
NedCor
SomCor
EcfCor
FoCor
GimCor
GzCor
NhCor
HjCor
TraCor
HpvCor
GlgCor
VaCor
VdCor
GgtCor
MagCor
GrcCor
CrpCor
TumCor
ShcCor
ShoCor
SpCor
SjCor
Ca_aCor
Ca_bCor
CadCor
Ct_aCor
CapCor
LoeCor
ShsCor
DehCor
MrgCor
CllCor
Pia_bCor
Pcp_aCor
Pcp_bCor
CglCor
NacCor
Sab_aCor
Sab_bCor
SakCor
SmiCor
Sc_aCor
Sc_bCor
Sc_cCor
Sap_aCor
VpCor
ZrCor
KaCor
KlCor
KlwCor
Lak_aCor
LatCor
LwCor
ErgCor
WaCor
YlCor
AlmCor_Aalpha
AlmCor_Abeta
AlmCor_Balpha
Bad_aCor
Bad_bCor
SppCor
MucCor
RhaCor_A
RhaCor_B
PhbCor
TctCor
CoCor
AimCor2A
CafCor2A
BtCor2A
OcCor2A
EqcCor2A
SsCor2A
Vertebrata
Class-2A
Metazoa
Class-2

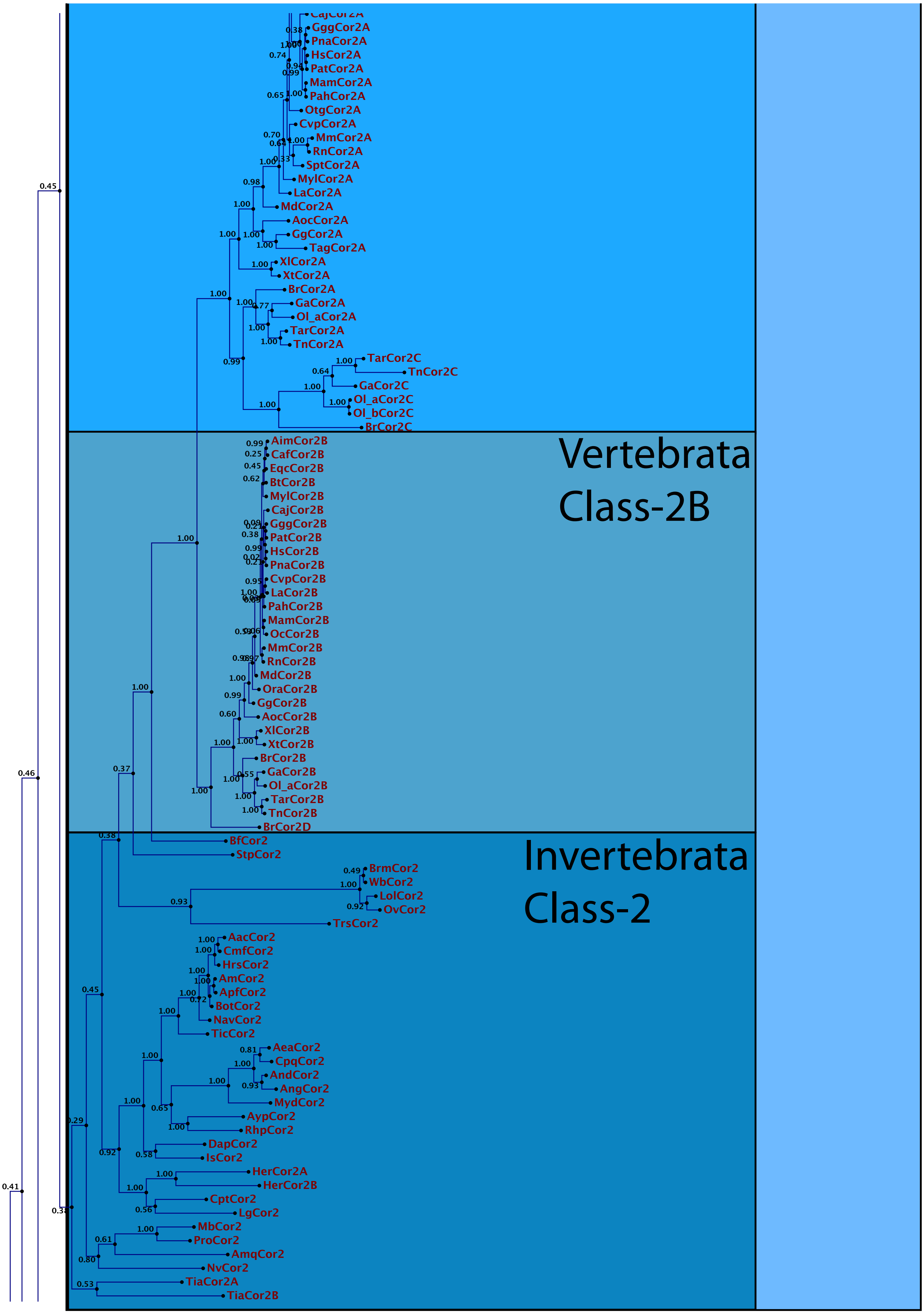
CajCor2A
GggCor2A
PnaCor2A
HsCor2A
PatCor2A
MamCor2A
PahCor2A
OtgCor2A
CvpCor2A
MmCor2A
RnCor2A
SptCor2A
MylCor2A
LaCor2A
MdCor2A
AocCor2A
GgCor2A
TagCor2A
XlCor2A
XtCor2A
BrCor2A
GaCor2A
Ol_aCor2A
TarCor2A
TnCor2A
TarCor2C
TnCor2C
GaCor2C
Ol_aCor2C
Ol_bCor2C
BrCor2C
AimCor2B
CafCor2B
EqcCor2B
BtCor2B
MylCor2B
CajCor2B
GggCor2B
PatCor2B
HsCor2B
PnaCor2B
CvpCor2B
LaCor2B
PahCor2B
MamCor2B
OcCor2B
MmCor2B
RnCor2B
MdCor2B
OraCor2B
GgCor2B
AocCor2B
XlCor2B
XtCor2B
BrCor2B
GaCor2B
Ol_aCor2B
TarCor2B
TnCor2B
BrCor2D
BfCor2
StpCor2
BrmCor2
WbCor2
LolCor2
OvCor2
TrsCor2
AacCor2
CmfCor2
HrsCor2
AmCor2
ApfCor2
BotCor2
NavCor2
TicCor2
AeaCor2
CpqCor2
AndCor2
AngCor2
MydCor2
AypCor2
RhpCor2
DapCor2
IsCor2
HerCor2A
HerCor2B
CptCor2
LgCor2
MbCor2
ProCor2
AmqCor2
NvCor2
TiaCor2A
TiaCor2B
Vertebrata
Class-2B
Invertebrata
Class-2

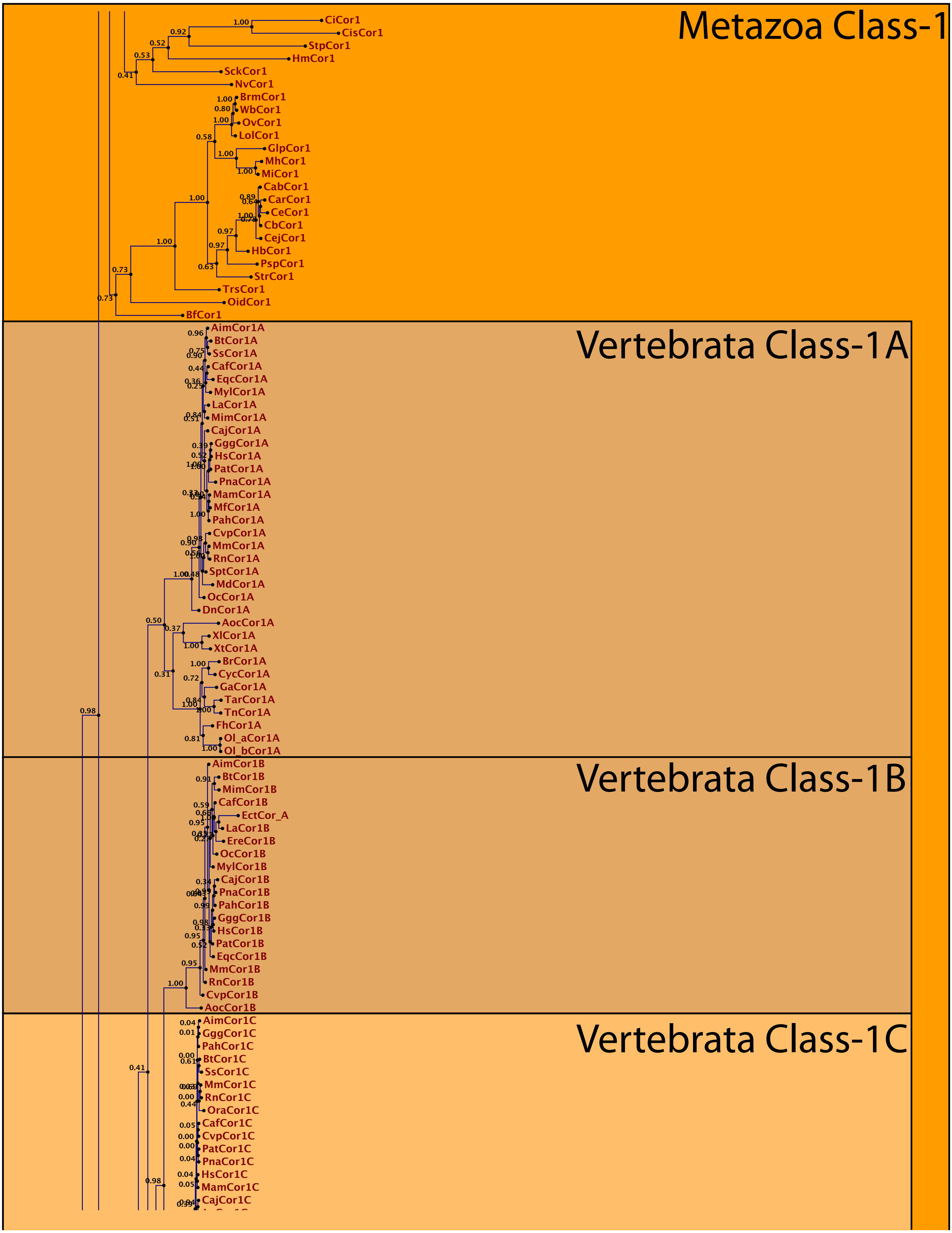

Metazoa Class-1
CiCor1
CisCor1
StpCor1
HmCor1
SckCor1
NvCor1
BrmCor1
WbCor1
OvCor1
LolCor1
GlpCor1
MhCor1
MiCor1
CabCor1
CarCor1
CeCor1
CbCor1
CejCor1
HbCor1
PspCor1
StrCor1
TrsCor1
OidCor1
BfCor1
Vertebrata Class-1A
AimCor1A
BtCor1A
SsCor1A
CafCor1A
EqcCor1A
MylCor1A
LaCor1A
MimCor1A
CajCor1A
GggCor1A
HsCor1A
PatCor1A
PnaCor1A
MamCor1A
MfCor1A
PahCor1A
CvpCor1A
MmCor1A
RnCor1A
SptCor1A
MdCor1A
OcCor1A
DnCor1A
AocCor1A
XlCor1A
XtCor1A
BrCor1A
CycCor1A
GaCor1A
TarCor1A
TnCor1A
FhCor1A
Ol_aCor1A
Ol_bCor1A
Vertebrata Class-1B
AimCor1B
BtCor1B
MimCor1B
CafCor1B
EctCor_A
LaCor1B
EreCor1B
OcCor1B
MylCor1B
CajCor1B
PnaCor1B
PahCor1B
GggCor1B
HsCor1B
PatCor1B
EqcCor1B
MmCor1B
RnCor1B
CvpCor1B
AocCor1B
Vertebrata Class-1C
AimCor1C
GggCor1C
PahCor1C
BtCor1C
SsCor1C
MmCor1C
RnCor1C
OraCor1C
CafCor1C
CvpCor1C
PatCor1C
PnaCor1C
HsCor1C
MamCor1C
CajCor1C

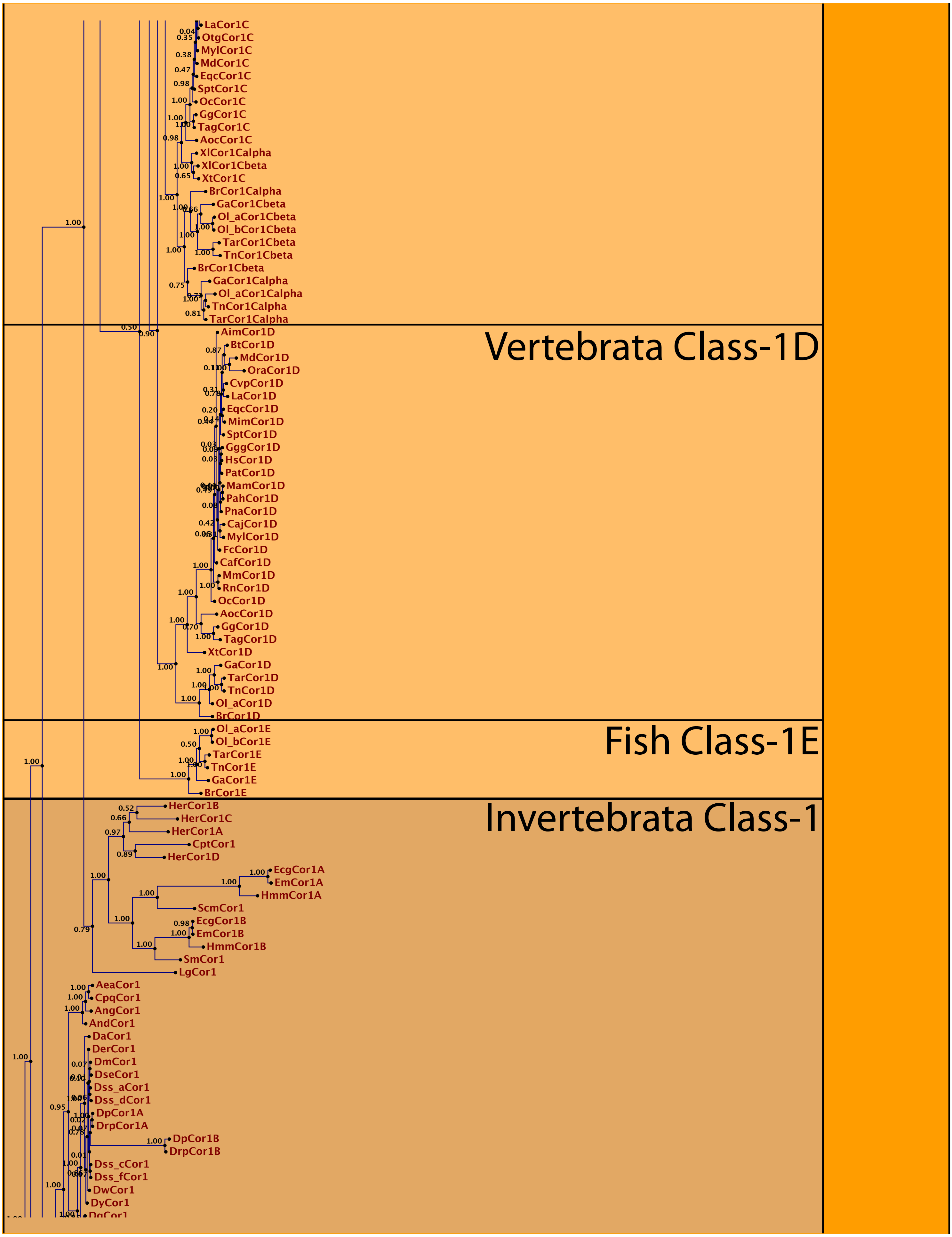
LaCor1C
OtgCor1C
MylCor1C
MdCor1C
EqcCor1C
SptCor1C
OcCor1C
GgCor1C
TagCor1C
AocCor1C
XlCor1Calpha
XlCor1Cbeta
XtCor1C
BrCor1Calpha
GaCor1Cbeta
Ol_aCor1Cbeta
Ol_bCor1Cbeta
TarCor1Cbeta
TnCor1Cbeta
BrCor1Cbeta
GaCor1Calpha
Ol_aCor1Calpha
TnCor1Calpha
TarCor1Calpha
Vertebrata Class-1D
AimCor1D
BtCor1D
MdCor1D
OraCor1D
CvpCor1D
LaCor1D
EqcCor1D
MimCor1D
SptCor1D
GggCor1D
HsCor1D
PatCor1D
MamCor1D
PahCor1D
PnaCor1D
CajCor1D
MylCor1D
FcCor1D
CafCor1D
MmCor1D
RnCor1D
OcCor1D
AocCor1D
GgCor1D
TagCor1D
XtCor1D
GaCor1D
TarCor1D
TnCor1D
Ol_aCor1D
BrCor1D
Fish Class-1E
Ol_aCor1E
Ol_bCor1E
TarCor1E
TnCor1E
GaCor1E
BrCor1E
Invertebrata Class-1
HerCor1B
HerCor1C
HerCor1A
CptCor1
HerCor1D
EcgCor1A
EmCor1A
HmmCor1A
ScmCor1
EcgCor1B
EmCor1B
HmmCor1B
SmCor1
LgCor1
AeaCor1
CpqCor1
AngCor1
AndCor1
DaCor1
DerCor1
DmCor1
DseCor1
Dss_aCor1
Dss_dCor1
DpCor1A
DrpCor1A
DpCor1B
DrpCor1B
Dss_cCor1
Dss_fCor1
DwCor1
DyCor1

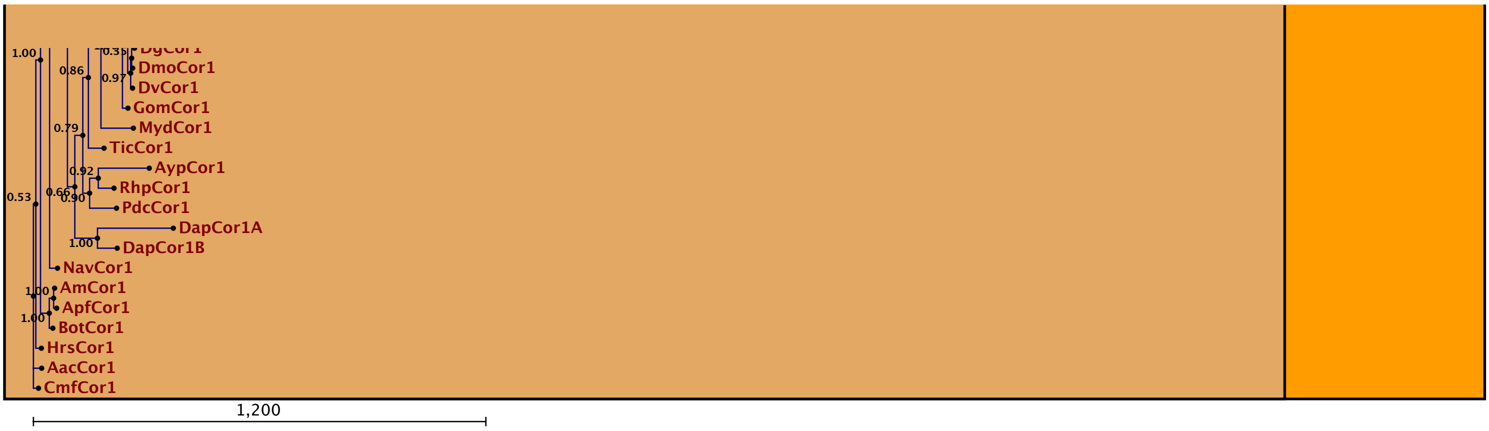
DmoCor1
DvCor1
GomCor1
MydCor1
TicCor1
AypCor1
RhpCor1
PdcCor1
DapCor1A
DapCor1B
NavCor1
AmCor1
ApfCor1
BotCor1
HrsCor1
AacCor1
CmfCor1
1,200
