# Supplementary material for: A holistic phylogeny of the coronin gene family reveals an ancient origin of the tandem-coronin, defines a new subfamily, and predicts protein function
Source: BMC Evol Biol. 2011 Sep 25;11:268. doi: 10.1186/1471-2148-11-268 (PMC3203266; doi:10.1186/1471-2148-11-268)
Supplement: Additional file 3 — RAxML tree of the coronin family This file contains the phylogenetic tree calculated with RAxML including bootstrap values. The tree is plotted in an extended way so that every coronin can be found and compared easily. [file 1471-2148-11-268-S3.PDF]

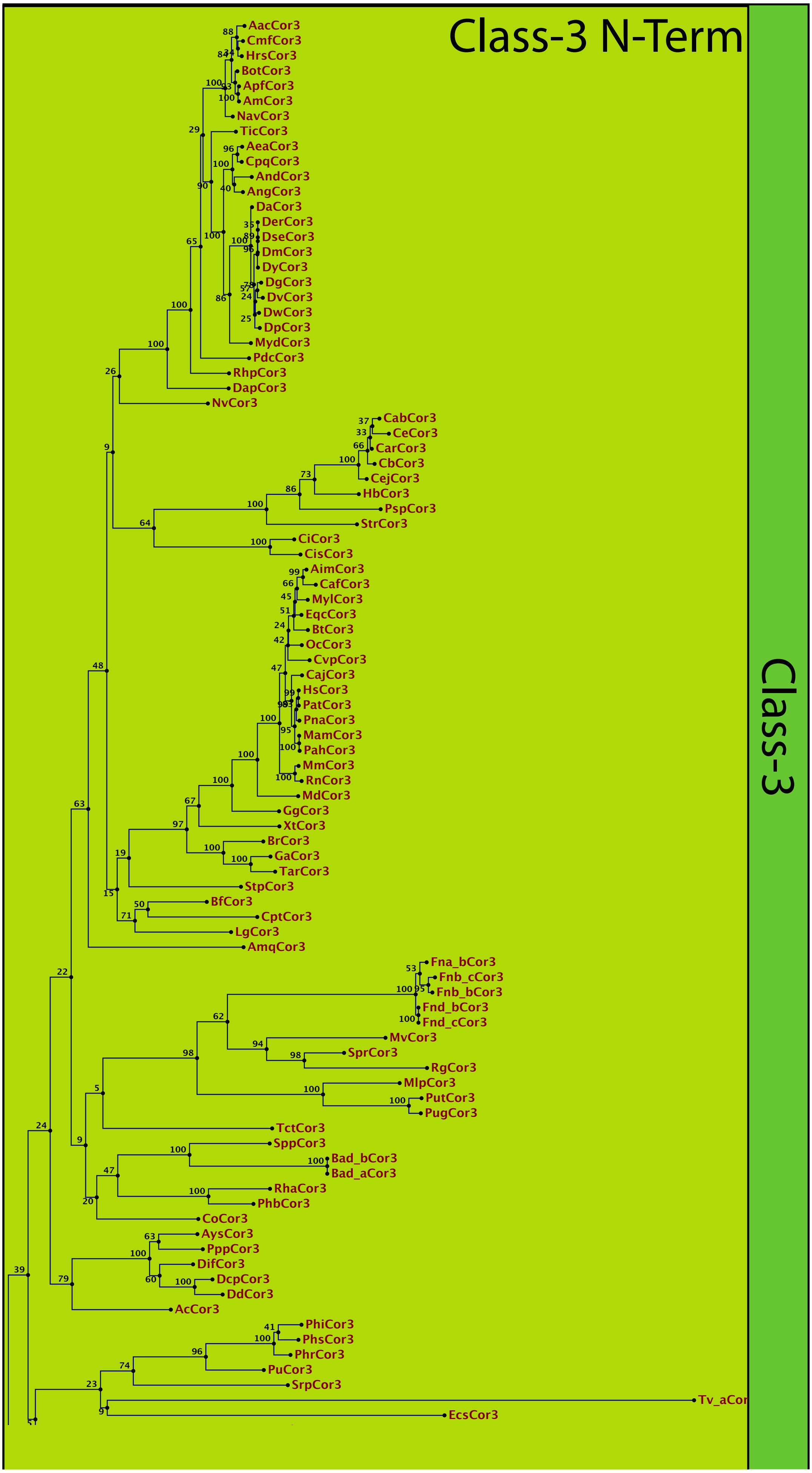
Class-3 N-Term
Class-3
AacCor3
CmfCor3
HrsCor3
BotCor3
ApfCor3
AmCor3
NavCor3
TicCor3
AeaCor3
CpqCor3
AndCor3
AngCor3
DaCor3
DerCor3
DseCor3
DmCor3
DyCor3
DgCor3
DvCor3
DwCor3
DpCor3
MydCor3
PdcCor3
RhpCor3
DapCor3
NvCor3
CabCor3
CeCor3
CarCor3
CbCor3
CejCor3
HbCor3
PspCor3
StrCor3
CiCor3
CisCor3
AimCor3
CafCor3
MylCor3
EqcCor3
BtCor3
OcCor3
CvpCor3
CajCor3
HsCor3
PatCor3
PnaCor3
MamCor3
PahCor3
MmCor3
RnCor3
MdCor3
GgCor3
XtCor3
BrCor3
GaCor3
TarCor3
StpCor3
BfCor3
CptCor3
LgCor3
AmqCor3
Fna_bCor3
Fnb_cCor3
Fnb_bCor3
Fnd_bCor3
Fnd_cCor3
MvCor3
SprCor3
RgCor3
MlpCor3
PutCor3
PugCor3
TctCor3
SppCor3
Bad_bCor3
Bad_aCor3
RhaCor3
PhbCor3
CoCor3
AysCor3
PppCor3
DifCor3
DcpCor3
DdCor3
AcCor3
PhiCor3
PhsCor3
PhrCor3
PuCor3
SrpCor3
Tv_aCor
EcsCor3

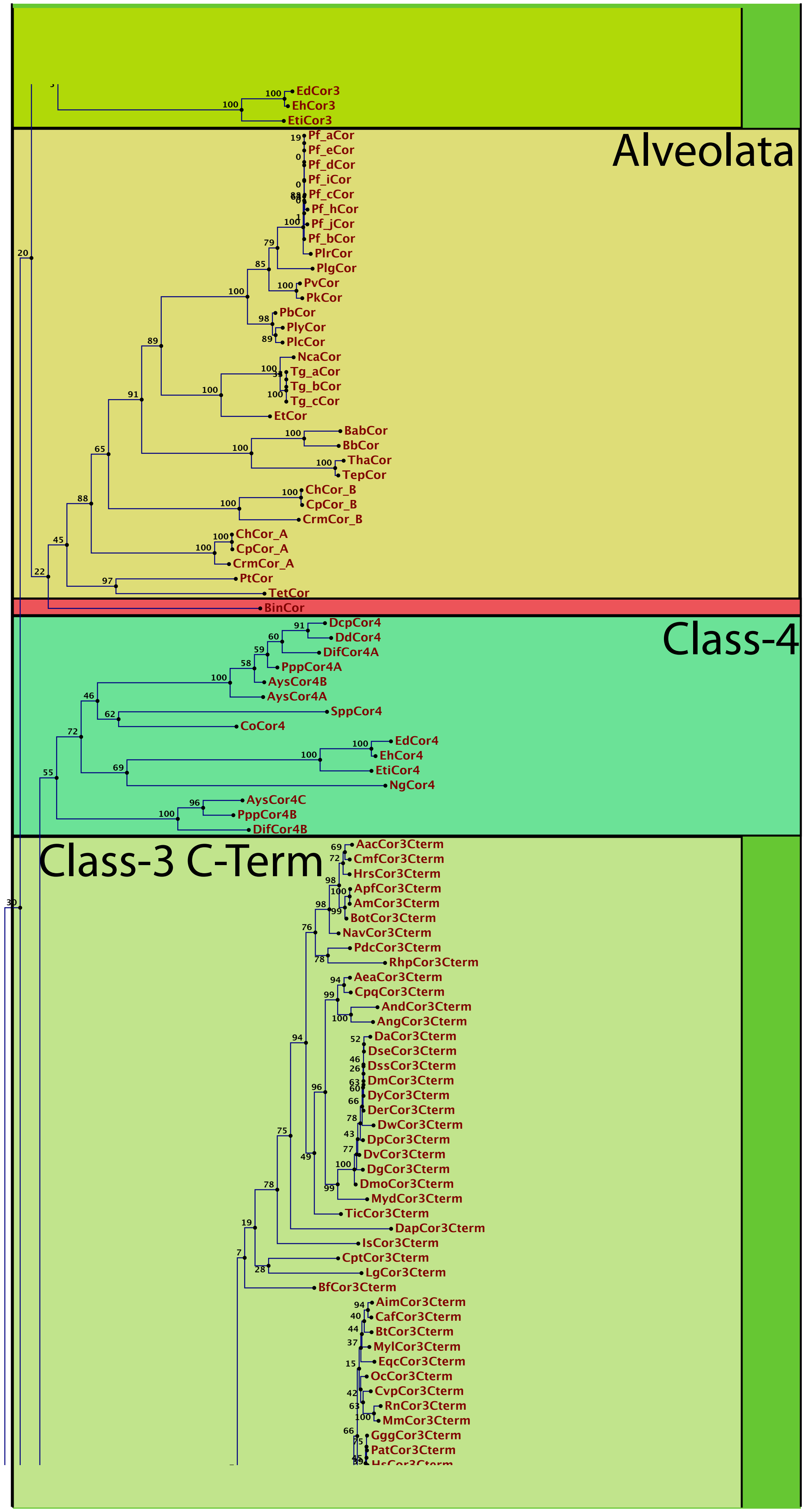
Alveolata
Class-4
Class-3 C-Term
EdCor3
EhCor3
EtiCor3
Pf_aCor
Pf_eCor
Pf_dCor
Pf_iCor
Pf_cCor
Pf_hCor
Pf_jCor
Pf_bCor
PlrCor
PlgCor
PvCor
PkCor
PbCor
PlyCor
PlcCor
NcaCor
Tg_aCor
Tg_bCor
Tg_cCor
EtCor
BabCor
BbCor
ThaCor
TepCor
ChCor_B
CpCor_B
CrmCor_B
ChCor_A
CpCor_A
CrmCor_A
PtCor
TetCor
BinCor
DcpCor4
DdCor4
DifCor4A
PppCor4A
AysCor4B
AysCor4A
SppCor4
CoCor4
EdCor4
EhCor4
EtiCor4
NgCor4
AysCor4C
PppCor4B
DifCor4B
AacCor3Cterm
CmfCor3Cterm
HrsCor3Cterm
ApfCor3Cterm
AmCor3Cterm
BotCor3Cterm
NavCor3Cterm
PdcCor3Cterm
RhpCor3Cterm
AeaCor3Cterm
CpqCor3Cterm
AndCor3Cterm
AngCor3Cterm
DaCor3Cterm
DseCor3Cterm
DssCor3Cterm
DmCor3Cterm
DyCor3Cterm
DerCor3Cterm
DwCor3Cterm
DpCor3Cterm
DvCor3Cterm
DgCor3Cterm
DmoCor3Cterm
MydCor3Cterm
TicCor3Cterm
DapCor3Cterm
IsCor3Cterm
CptCor3Cterm
LgCor3Cterm
BfCor3Cterm
AimCor3Cterm
CafCor3Cterm
BtCor3Cterm
MylCor3Cterm
EqcCor3Cterm
OcCor3Cterm
CvpCor3Cterm
RnCor3Cterm
MmCor3Cterm
GggCor3Cterm
PatCor3Cterm

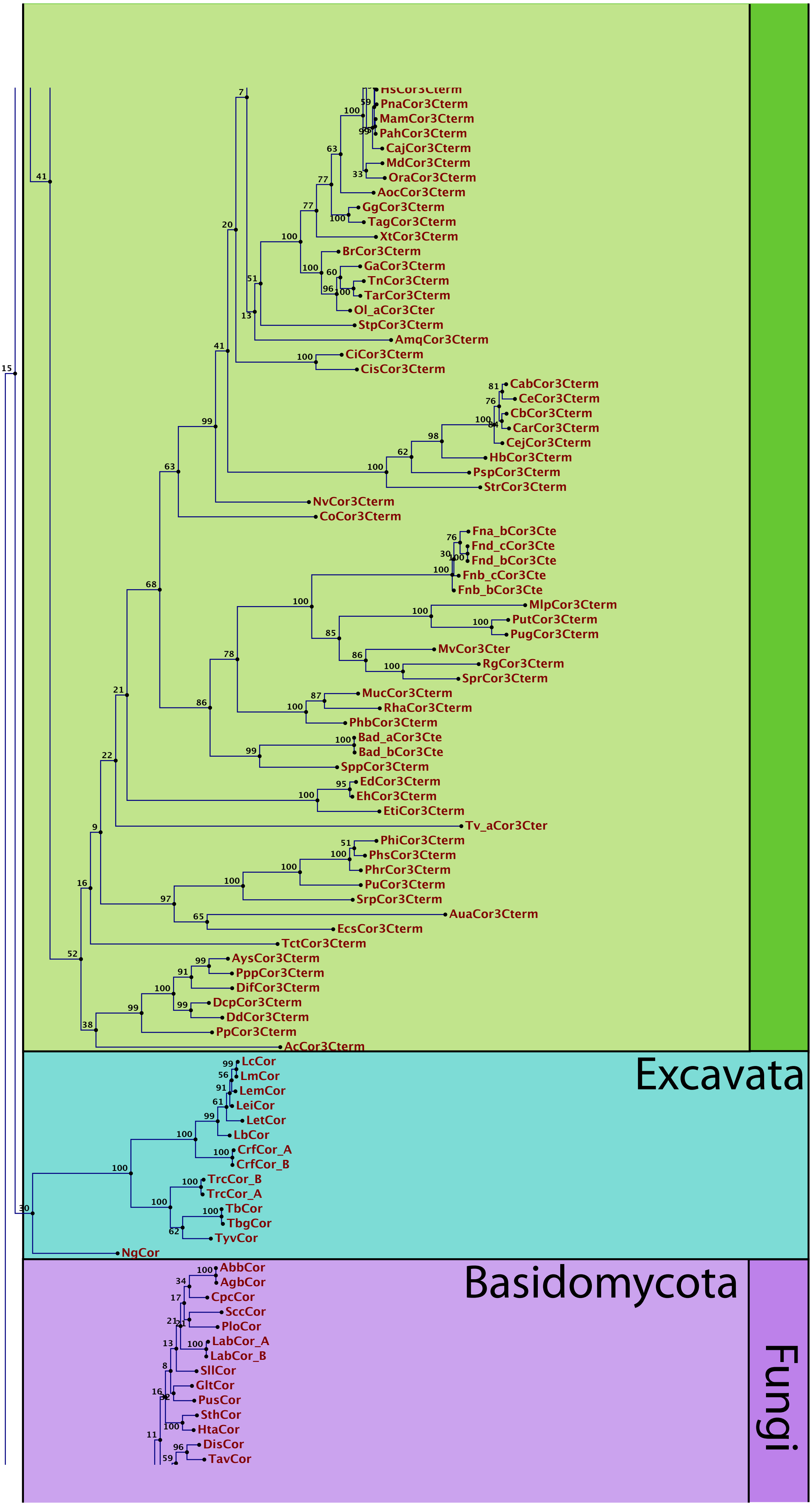
PnaCor3Cterm
MamCor3Cterm
PahCor3Cterm
CajCor3Cterm
MdCor3Cterm
OraCor3Cterm
AocCor3Cterm
GgCor3Cterm
TagCor3Cterm
XtCor3Cterm
BrCor3Cterm
GaCor3Cterm
TnCor3Cterm
TarCor3Cterm
Ol_aCor3Cter
StpCor3Cterm
AmqCor3Cterm
CiCor3Cterm
CisCor3Cterm
CabCor3Cterm
CeCor3Cterm
CbCor3Cterm
CarCor3Cterm
CejCor3Cterm
HbCor3Cterm
PspCor3Cterm
StrCor3Cterm
NvCor3Cterm
CoCor3Cterm
Fna_bCor3Cte
Fnd_cCor3Cte
Fnd_bCor3Cte
Fnb_cCor3Cte
Fnb_bCor3Cte
MlpCor3Cterm
PutCor3Cterm
PugCor3Cterm
MvCor3Cter
RgCor3Cterm
SprCor3Cterm
MucCor3Cterm
RhaCor3Cterm
PhbCor3Cterm
Bad_aCor3Cte
Bad_bCor3Cte
SppCor3Cterm
EdCor3Cterm
EhCor3Cterm
EtiCor3Cterm
Tv_aCor3Cter
PhiCor3Cterm
PhsCor3Cterm
PhrCor3Cterm
PuCor3Cterm
SrpCor3Cterm
AuaCor3Cterm
EcsCor3Cterm
TctCor3Cterm
AysCor3Cterm
PppCor3Cterm
DifCor3Cterm
DcpCor3Cterm
DdCor3Cterm
PpCor3Cterm
AcCor3Cterm
Excavata
LcCor
LmCor
LemCor
LeiCor
LetCor
LbCor
CrfCor_A
CrfCor_B
TrcCor_B
TrcCor_A
TbCor
TbgCor
TyvCor
NgCor
Basidomycota
Fungi
AbbCor
AgbCor
CpcCor
SccCor
PloCor
LabCor_A
LabCor_B
SllCor
GltCor
PusCor
SthCor
HtaCor
DisCor
TavCor

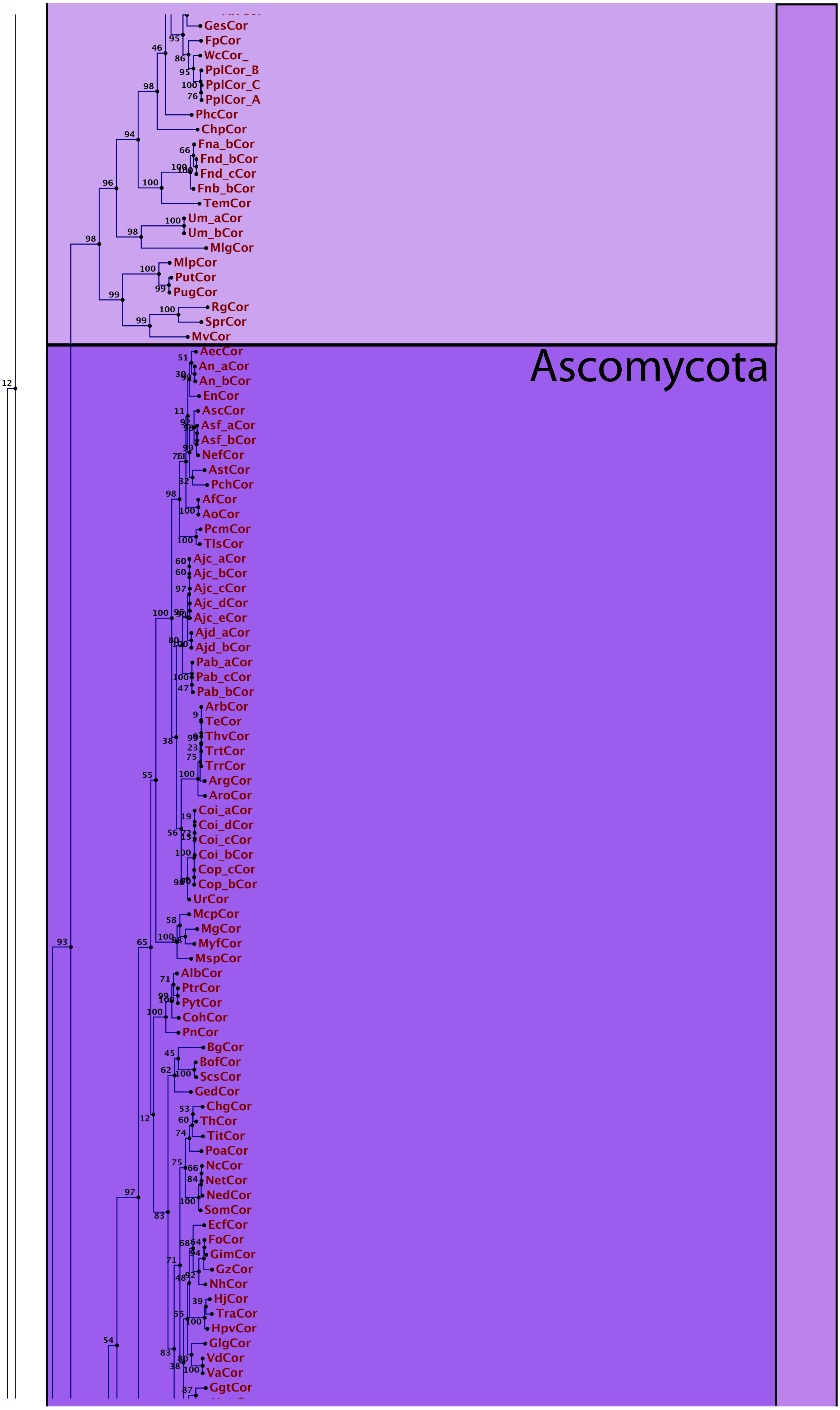
Ascomycota
GesCor
FpCor
WcCor_
PplCor_B
PplCor_C
PplCor_A
PhcCor
ChpCor
Fna_bCor
Fnd_bCor
Fnd_cCor
Fnb_bCor
TemCor
Um_aCor
Um_bCor
MlgCor
MlpCor
PutCor
PugCor
RgCor
SprCor
MvCor
AecCor
An_aCor
An_bCor
EnCor
AscCor
Asf_aCor
Asf_bCor
NefCor
AstCor
PchCor
AfCor
AoCor
PcmCor
TlsCor
Ajc_aCor
Ajc_bCor
Ajc_cCor
Ajc_dCor
Ajc_eCor
Ajd_aCor
Ajd_bCor
Pab_aCor
Pab_cCor
Pab_bCor
ArbCor
TeCor
ThvCor
TrtCor
TrrCor
ArgCor
AroCor
Coi_aCor
Coi_dCor
Coi_cCor
Coi_bCor
Cop_cCor
Cop_bCor
UrCor
McpCor
MgCor
MyfCor
MspCor
AlbCor
PtrCor
PytCor
CohCor
PnCor
BgCor
BofCor
ScsCor
GedCor
ChgCor
ThCor
TitCor
PoaCor
NcCor
NetCor
NedCor
SomCor
EcfCor
FoCor
GimCor
GzCor
NhCor
HjCor
TraCor
HpvCor
GlgCor
VdCor
VaCor
GgtCor

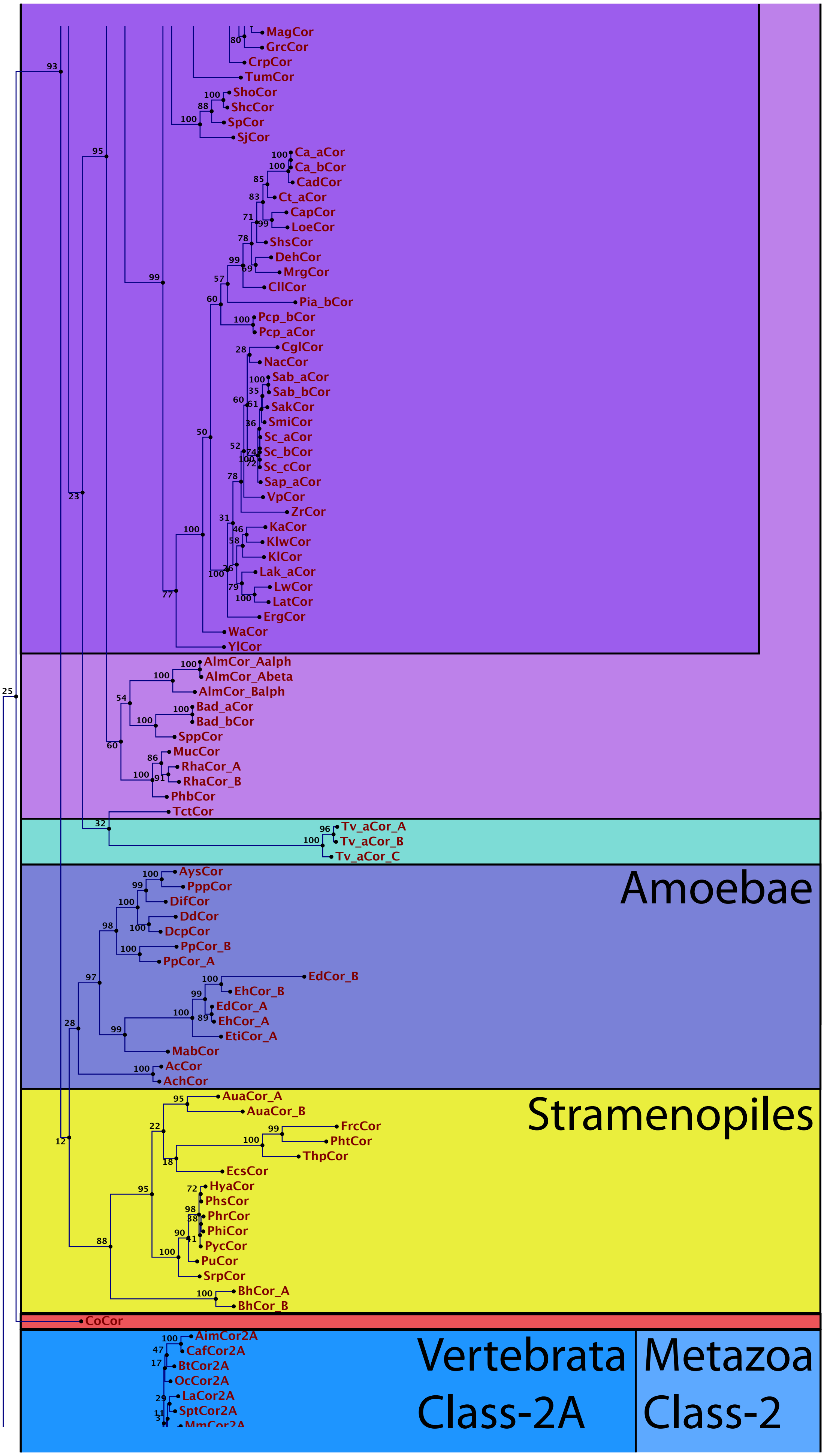
MagCor
GrcCor
CrpCor
TumCor
ShoCor
ShcCor
SpCor
SjCor
Ca_aCor
Ca_bCor
CadCor
Ct_aCor
CapCor
LoeCor
ShsCor
DehCor
MrgCor
CllCor
Pia_bCor
Pcp_bCor
Pcp_aCor
CglCor
NacCor
Sab_aCor
Sab_bCor
SakCor
SmiCor
Sc_aCor
Sc_bCor
Sc_cCor
Sap_aCor
VpCor
ZrCor
KaCor
KlwCor
KlCor
Lak_aCor
LwCor
LatCor
ErgCor
WaCor
YlCor
AlmCor_Aalph
AlmCor_Abeta
AlmCor_Balph
Bad_aCor
Bad_bCor
SppCor
MucCor
RhaCor_A
RhaCor_B
PhbCor
TctCor
Tv_aCor_A
Tv_aCor_B
Tv_aCor_C
Amoebae
AysCor
PppCor
DifCor
DdCor
DcpCor
PpCor_B
PpCor_A
EdCor_B
EhCor_B
EdCor_A
EhCor_A
EtiCor_A
MabCor
AcCor
AchCor
Stramenopiles
AuaCor_A
AuaCor_B
FrcCor
PhtCor
ThpCor
EcsCor
HyaCor
PhsCor
PhrCor
PhiCor
PycCor
PuCor
SrpCor
BhCor_A
BhCor_B
CoCor
AimCor2A
CafCor2A
BtCor2A
OcCor2A
LaCor2A
SptCor2A
Vertebrata Class-2A
Metazoa Class-2

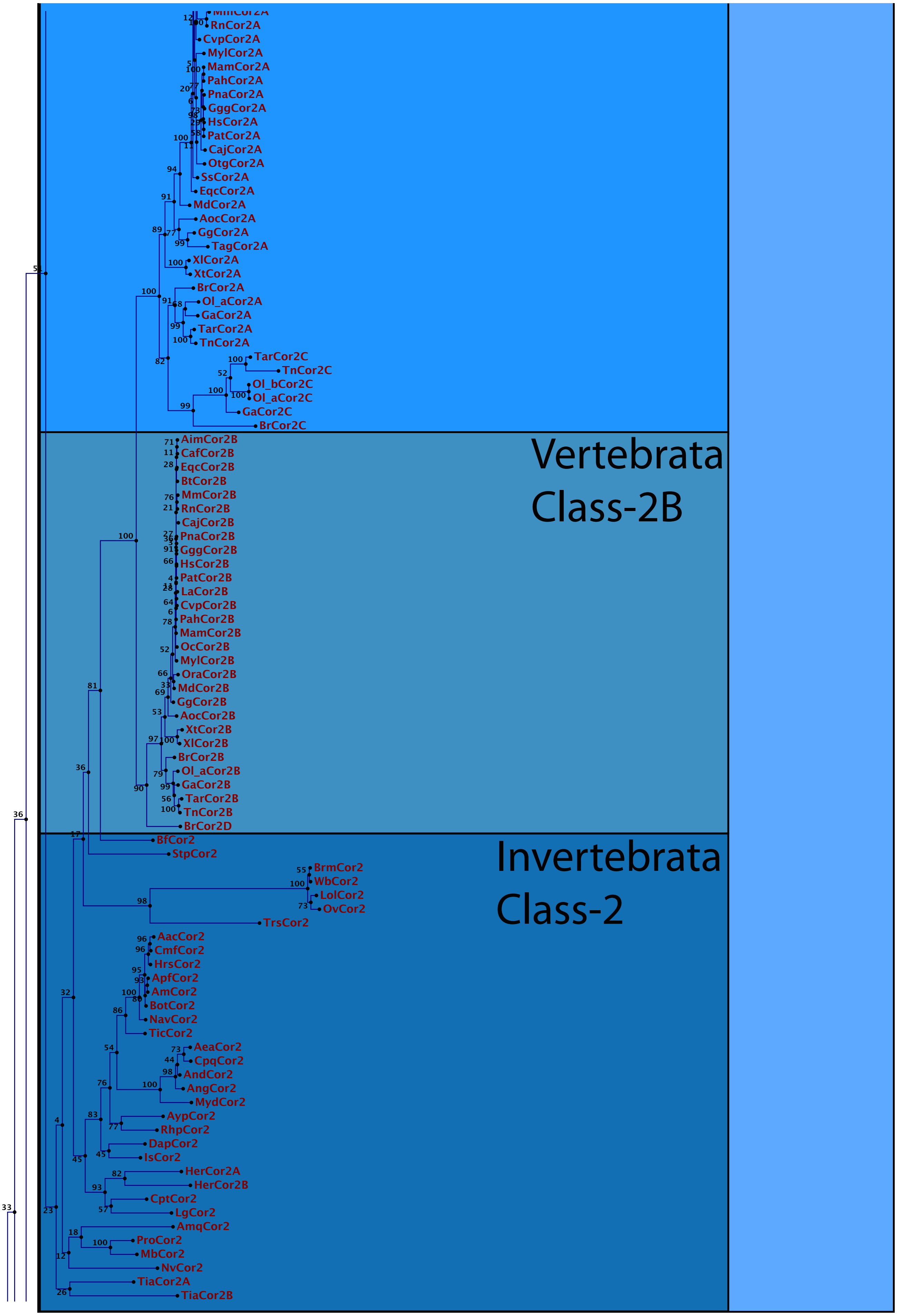
RnCor2A
CvpCor2A
MylCor2A
MamCor2A
PahCor2A
PnaCor2A
GggCor2A
HsCor2A
PatCor2A
CajCor2A
OtgCor2A
SsCor2A
EqcCor2A
MdCor2A
AocCor2A
GgCor2A
TagCor2A
XlCor2A
XtCor2A
BrCor2A
Ol_aCor2A
GaCor2A
TarCor2A
TnCor2A
TarCor2C
TnCor2C
Ol_bCor2C
Ol_aCor2C
GaCor2C
BrCor2C
Vertebrata
Class-2B
AimCor2B
CafCor2B
EqcCor2B
BtCor2B
MmCor2B
RnCor2B
CajCor2B
PnaCor2B
GggCor2B
HsCor2B
PatCor2B
LaCor2B
CvpCor2B
PahCor2B
MamCor2B
OcCor2B
MylCor2B
OraCor2B
MdCor2B
GgCor2B
AocCor2B
XtCor2B
XlCor2B
BrCor2B
Ol_aCor2B
GaCor2B
TarCor2B
TnCor2B
BrCor2D
Invertebrata
Class-2
BfCor2
StpCor2
BrmCor2
WbCor2
LolCor2
OvCor2
TrsCor2
AacCor2
CmfCor2
HrsCor2
ApfCor2
AmCor2
BotCor2
NavCor2
TicCor2
AeaCor2
CpqCor2
AndCor2
AngCor2
MydCor2
AypCor2
RhpCor2
DapCor2
IsCor2
HerCor2A
HerCor2B
CptCor2
LgCor2
AmqCor2
ProCor2
MbCor2
NvCor2
TiaCor2A
TiaCor2B

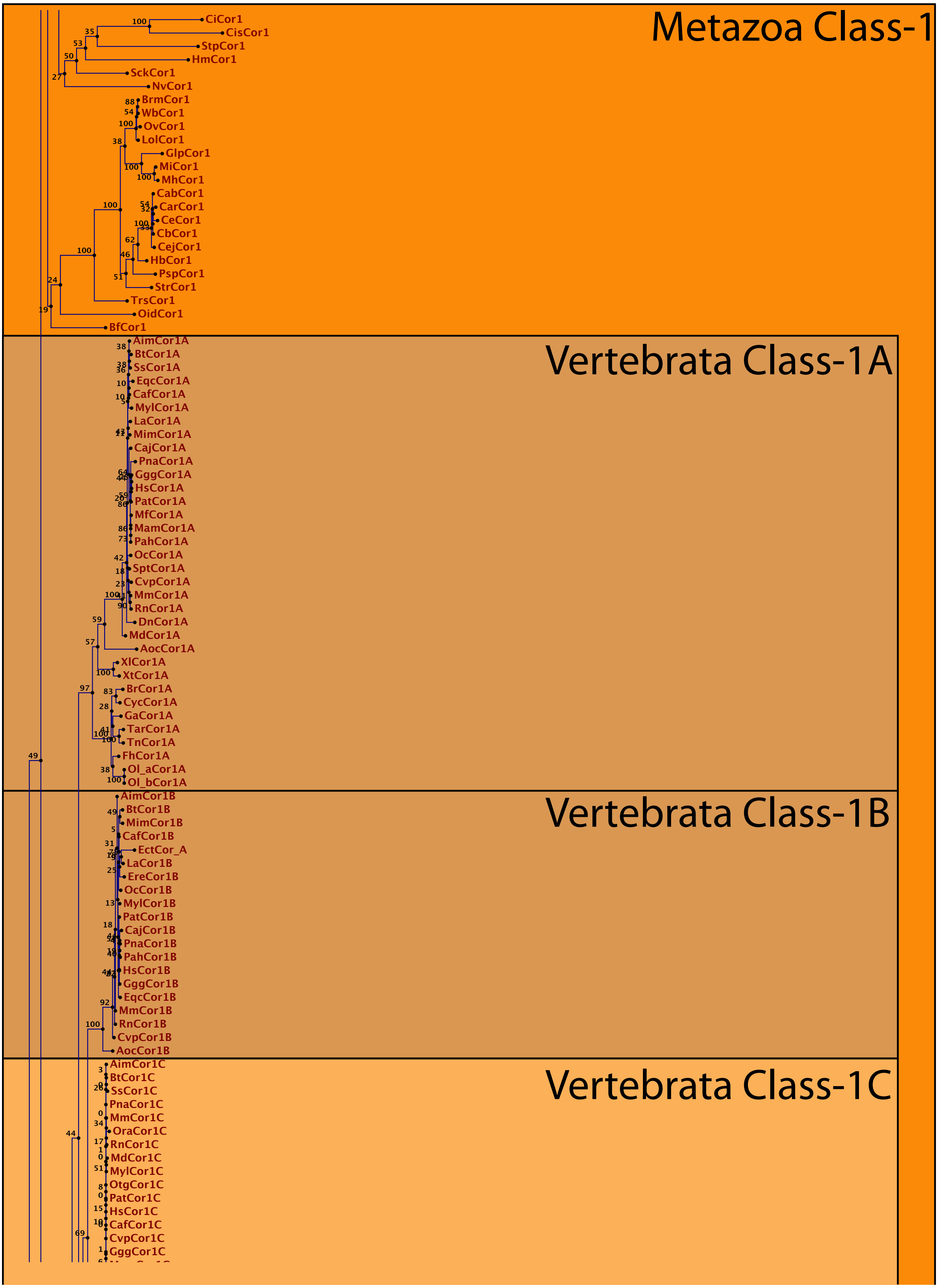
Metazoa Class-1
CiCor1
CisCor1
StpCor1
HmCor1
SckCor1
NvCor1
BrmCor1
WbCor1
OvCor1
LolCor1
GlpCor1
MiCor1
MhCor1
CabCor1
CarCor1
CeCor1
CbCor1
CejCor1
HbCor1
PspCor1
StrCor1
TrsCor1
OidCor1
BfCor1
Vertebrata Class-1A
AimCor1A
BtCor1A
SsCor1A
EqcCor1A
CafCor1A
MylCor1A
LaCor1A
MimCor1A
CajCor1A
PnaCor1A
GggCor1A
HsCor1A
PatCor1A
MfCor1A
MamCor1A
PahCor1A
OcCor1A
SptCor1A
CvpCor1A
MmCor1A
RnCor1A
DnCor1A
MdCor1A
AocCor1A
XlCor1A
XtCor1A
BrCor1A
CycCor1A
GaCor1A
TarCor1A
TnCor1A
FhCor1A
Ol_aCor1A
Ol_bCor1A
Vertebrata Class-1B
AimCor1B
BtCor1B
MimCor1B
CafCor1B
EctCor_A
LaCor1B
EreCor1B
OcCor1B
MylCor1B
PatCor1B
CajCor1B
PnaCor1B
PahCor1B
HsCor1B
GggCor1B
EqcCor1B
MmCor1B
RnCor1B
CvpCor1B
AocCor1B
Vertebrata Class-1C
AimCor1C
BtCor1C
SsCor1C
PnaCor1C
MmCor1C
OraCor1C
RnCor1C
MdCor1C
MylCor1C
OtgCor1C
PatCor1C
HsCor1C
CafCor1C
CvpCor1C
GggCor1C

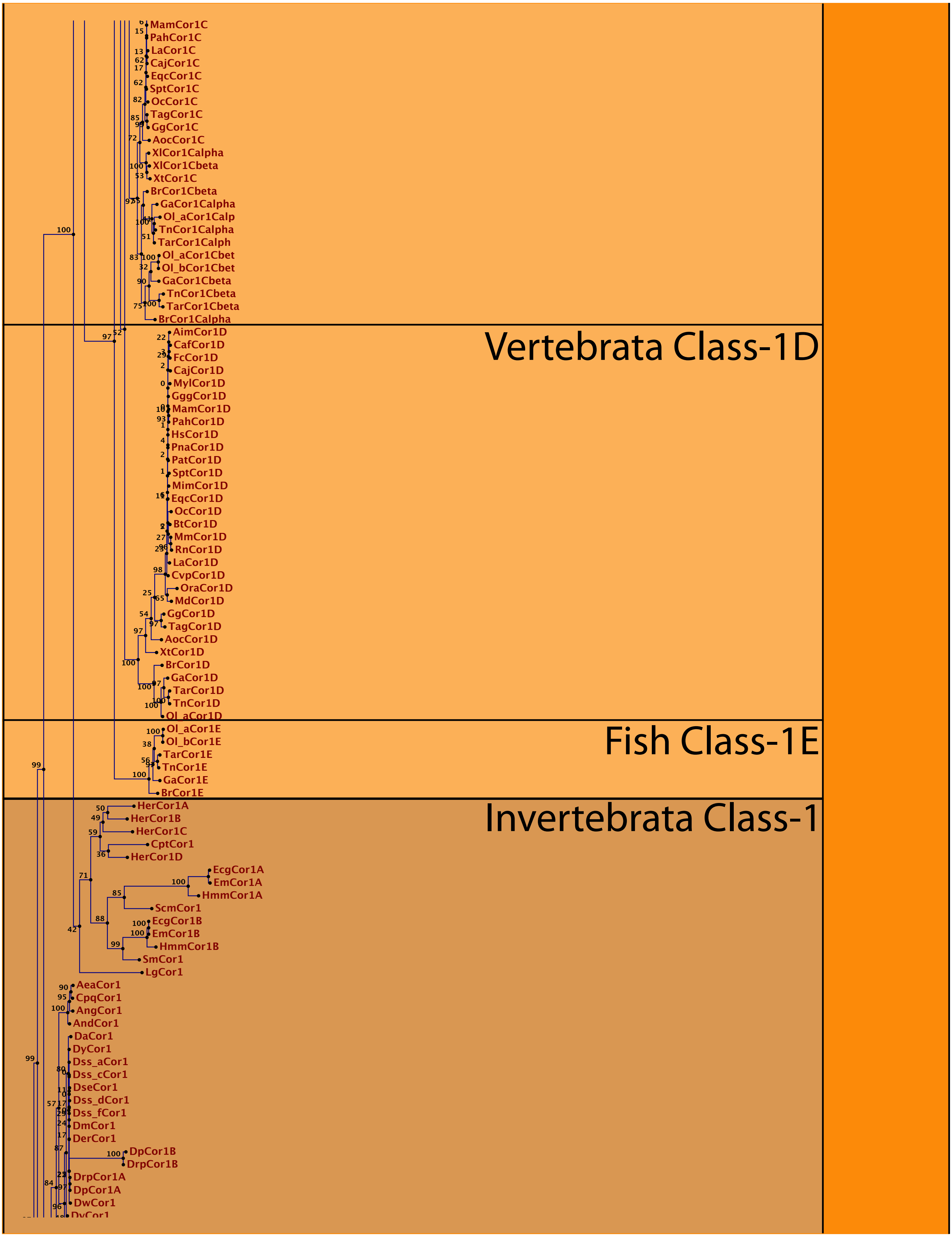
MamCor1C
PahCor1C
LaCor1C
CajCor1C
EqcCor1C
SptCor1C
OcCor1C
TagCor1C
GgCor1C
AocCor1C
XlCor1Calpha
XlCor1Cbeta
XtCor1C
BrCor1Cbeta
GaCor1Calpha
Ol_aCor1Calp
TnCor1Calpha
TarCor1Calph
Ol_aCor1Cbet
Ol_bCor1Cbet
GaCor1Cbeta
TnCor1Cbeta
TarCor1Cbeta
BrCor1Calpha
Vertebrata Class-1D
AimCor1D
CafCor1D
FcCor1D
CajCor1D
MylCor1D
GggCor1D
MamCor1D
PahCor1D
HsCor1D
PnaCor1D
PatCor1D
SptCor1D
MimCor1D
EqcCor1D
OcCor1D
BtCor1D
MmCor1D
RnCor1D
LaCor1D
CvpCor1D
OraCor1D
MdCor1D
GgCor1D
TagCor1D
AocCor1D
XtCor1D
BrCor1D
GaCor1D
TarCor1D
TnCor1D
Ol_aCor1D
Fish Class-1E
Ol_aCor1E
Ol_bCor1E
TarCor1E
TnCor1E
GaCor1E
BrCor1E
Invertebrata Class-1
HerCor1A
HerCor1B
HerCor1C
CptCor1
HerCor1D
EcgCor1A
EmCor1A
HmmCor1A
ScmCor1
EcgCor1B
EmCor1B
HmmCor1B
SmCor1
LgCor1
AeaCor1
CpqCor1
AngCor1
AndCor1
DaCor1
DyCor1
Dss_aCor1
Dss_cCor1
DseCor1
Dss_dCor1
Dss_fCor1
DmCor1
DerCor1
DpCor1B
DrpCor1B
DrpCor1A
DpCor1A
DwCor1

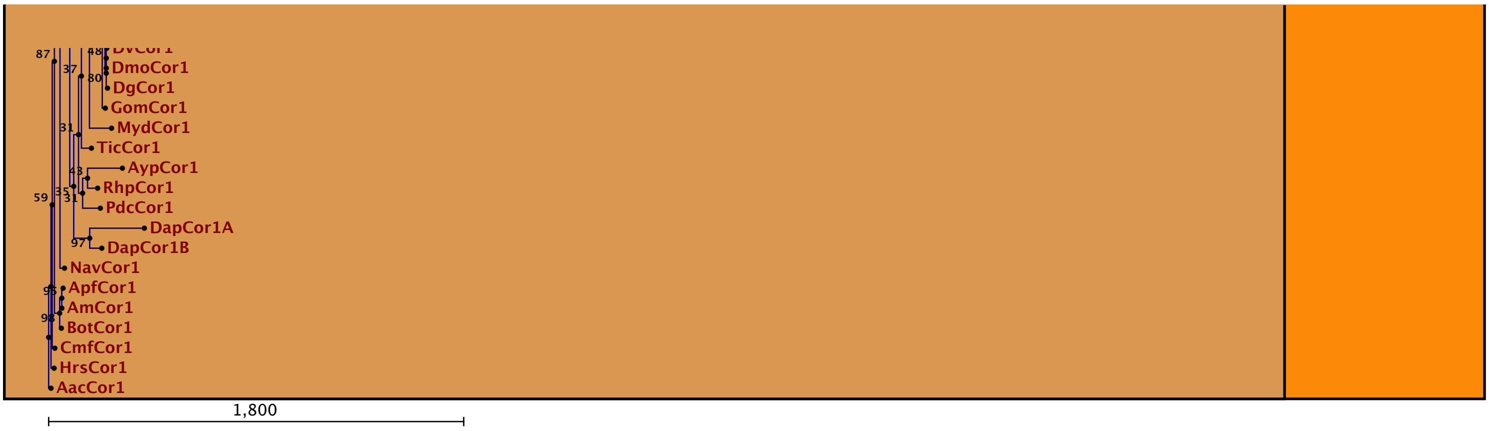
87
48
DmoCor1
37
80
DgCor1
GomCor1
MydCor1
31
TicCor1
43
AypCor1
59
35
31
RhpCor1
PdcCor1
DapCor1A
97
DapCor1B
NavCor1
ApfCor1
AmCor1
BotCor1
CmfCor1
HrsCor1
AacCor1
1,800
