# Supplementary material for: A holistic phylogeny of the coronin gene family reveals an ancient origin of the tandem-coronin, defines a new subfamily, and predicts protein function
Source: BMC Evol Biol. 2011 Sep 25;11:268. doi: 10.1186/1471-2148-11-268 (PMC3203266; doi:10.1186/1471-2148-11-268)
Supplement: Additional file 4 — Coronin repertoire of all eukaryotes analyzed Complete table of the coronin inventories of 358 eukaryotes. [file 1471-2148-11-268-S4.PDF]

Coronin

cellular organisms	Compl.	No. Class	No. Seq	1	2	3	4	Or	
M. balamuthi [?]	?	0	1	-	-	-	-	1	-
A. castellanii [?]	?	1	2	-	-	1	-	1	-
A. healyi [?]	?	0	1	-	-	-	-	1	-
P. polycephalum [?]	?	1	3	-	-	1	-	2	-
H. vermiformis [?]	?	0	1	-	-	-	-	1	-
T. trahens [?]	?	1	2	-	-	1	-	1	-
N. gruberi [?]	?	1	2	-	-	-	1	1	-
M. ovata [?]	?	1	1	-	1	-	-	-	-
M. brevicollis [?]	?	1	1	-	1	-	-	-	-
P. sp. [?]	?	1	1	-	1	-	-	-	-
C. owczarzaki [?]	?	2	3	-	-	1	1	1	-
T. vaginalis [?]	?	1	4	-	-	1	-	3	-
B. natans [?]	?	0	1	-	-	-	-	1	-

Alveolata	Compl.	No. Class	No. Seq	1	2	3	4	Or	
T. thermophila [?]	?	0	1	-	-	-	-	1	-
P. tetraurelia [?]	?	0	1	-	-	-	-	1	-

Apicomplexa	Compl.	No. Class	No. Seq	1	2	3	4	Or	
B. bigemina [?]	?	0	1	-	-	-	-	1	-
B. bovis [?]	?	0	1	-	-	-	-	1	-
T. annulata [?]	?	0	1	-	-	-	-	1	-
T. parva [?]	?	0	1	-	-	-	-	1	-
C. parvum [?]	?	0	2	-	-	-	-	2	-
C. hominis [?]	?	0	2	-	-	-	-	2	-
C. muris [?]	?	0	2	-	-	-	-	2	-
E. tenella [?]	?	0	1	-	-	-	-	1	-
N. caninum [?]	?	0	1	-	-	-	-	1	-
T. gondii [?]	?	0	1	-	-	-	-	1	-
T. gondii [?]	?	0	1	-	-	-	-	1	-
T. gondii [?]	?	0	1	-	-	-	-	1	-

Haemosporida	Compl.	No. Class	No. Seq	1	2	3	4	Or	
P. gallinaceum [?]	?	0	1	-	-	-	-	1	-
P. falciparum [?]	?	0	1	-	-	-	-	1	-
P. falciparum [?]	?	0	1	-	-	-	-	1	-
P. reichenowi [?]	?	0	1	-	-	-	-	1	-
P. falciparum [?]	?	0	1	-	-	-	-	1	-
P. falciparum [?]	?	0	1	-	-	-	-	1	-
P. falciparum [?]	?	0	1	-	-	-	-	1	-
P. falciparum [?]	?	0	1	-	-	-	-	1	-
P. falciparum [?]	?	0	1	-	-	-	-	1	-
P. falciparum [?]	?	0	1	-	-	-	-	1	-
P. falciparum [?]	?	0	1	-	-	-	-	1	-
P. falciparum [?]	?	0	1	-	-	-	-	1	-
P. falciparum [?]	?	0	1	-	-	-	-	1	-
P. falciparum [?]	?	0	1	-	-	-	-	1	-

P. falciparum [?]	?	0	1	-	-	-	-	1	-
P. falciparum [?]	?	0	1	-	-	-	-	1	-
P. knowlesi [?]	?	0	1	-	-	-	-	1	-
P. vivax [?]	?	0	1	-	-	-	-	1	-
P. chabaudi [?]	?	0	1	-	-	-	-	1	-
P. berghei [?]	?	0	1	-	-	-	-	1	-
P. yoelii [?]	?	0	1	-	-	-	-	1	-

Entamoebidae	Compl.	No. Class	No. Seq	1	2	3	4	Or	
E. histolytica [?]	?	2	4	-	-	1	1	2	-
E. dispar [?]	?	2	3	-	-	1	1	1	1
E. invadens [?]	?	2	3	-	-	1	1	1	-

Dictyosteliida	Compl.	No. Class	No. Seq	1	2	3	4	Or	
A. subglobosum [?]	?	2	5	-	-	1	3	1	-
D. fasciculatum [?]	?	2	4	-	-	1	2	1	-
D. purpureum [?]	?	2	3	-	-	1	1	1	-
D. discoideum [?]	?	2	3	-	-	1	1	1	-
P. pallidum [?]	?	2	4	-	-	1	2	1	-

Euglenozoa	Compl.	No. Class	No. Seq	1	2	3	4	Or	
C. fasciculata [?]	?	0	2	-	-	-	-	2	-
L. donovani [?]	?	0	1	-	-	-	-	1	-
L. infantum [?]	?	0	1	-	-	-	-	1	-
L. major [?]	?	0	1	-	-	-	-	1	-
L. mexicana [?]	?	0	1	-	-	-	-	1	-
L. tarentolae [?]	?	0	1	-	-	-	-	1	-
L. braziliensis [?]	?	0	1	-	-	-	-	1	-
T. vivax [?]	?	0	1	-	-	-	-	1	-
T. congolense [?]	?	0	1	-	-	-	-	1	-
T. cruzi [?]	?	0	2	-	-	-	-	2	-
T. brucei [?]	?	0	1	-	-	-	-	1	-
T. brucei [?]	?	0	1	-	-	-	-	1	-

Fungi	Compl.	No. Class	No. Seq	1	2	3	4	Or	
A. macrogynus [?]	?	0	3	-	-	-	-	3	-
M. circinelloides [?]	?	1	2	-	-	1	-	1	-
P. blakesleeanus [?]	?	1	2	-	-	1	-	1	-
R. arrhizus [?]	?	1	3	-	-	1	-	2	-

Chytridiomycota	Compl.	No. Class	No. Seq	1	2	3	4	Or	
B. dendrobatidis [?]	?	1	2	-	-	1	-	1	-
B. dendrobatidis [?]	?	1	2	-	-	1	-	1	-
S. punctatus [?]	?	2	3	-	-	1	1	1	-

Ascomycota	Compl.	No. Class	No. Seq	1	2	3	4	Or	
M. fijiensis [?]	?	0	1	-	-	-	-	1	-
M. pini [?]	?	0	1	-	-	-	-	1	-
M. graminicola [?]	?	0	1	-	-	-	-	1	-
M. populorum [?]	?	0	1	-	-	-	-	1	-

P. nodorum [?]	?	0	1	-	-	-	-	1	-
C. heterostrophus [?]	?	0	1	-	-	-	-	1	-
A. brassicicola [?]	?	0	1	-	-	-	-	1	-
P. teres [?]	?	0	1	-	-	-	-	1	-
P. tritici-repentis [?]	?	0	1	-	-	-	-	1	-
B. graminis [?]	?	0	1	-	-	-	-	1	-
B. fuckeliana [?]	?	0	1	-	-	-	-	1	-
S. sclerotiorum [?]	?	0	1	-	-	-	-	1	-
G. destructans [?]	?	0	1	-	-	-	-	1	-
G. graminicola [?]	?	0	1	-	-	-	-	1	-
V. albo-atrum [?]	?	0	1	-	-	-	-	1	-
V. dahliae [?]	?	0	1	-	-	-	-	1	-
E. festucae [?]	?	0	1	-	-	-	-	1	-
H. jecorina [?]	?	0	1	-	-	-	-	1	-
H. virens [?]	?	0	1	-	-	-	-	1	-
T. atroviride [?]	?	0	1	-	-	-	-	1	-
F. oxysporum [?]	?	0	1	-	-	-	-	1	-
G. moniliformis [?]	?	0	1	-	-	-	-	1	-
G. zeae [?]	?	0	1	-	-	-	-	1	-
N. haematococca [?]	?	0	1	-	-	-	-	1	-
C. parasitica [?]	?	0	1	-	-	-	-	1	-
G. graminis [?]	?	0	1	-	-	-	-	1	-
M. grisea [?]	?	0	1	-	-	-	-	1	-
G. clavigera [?]	?	0	1	-	-	-	-	1	-
C. globosum [?]	?	0	1	-	-	-	-	1	-
T. heterothallica [?]	?	0	1	-	-	-	-	1	-
T. terrestris [?]	?	0	1	-	-	-	-	1	-
P. anserina [?]	?	0	1	-	-	-	-	1	-
N. crassa [?]	?	0	1	-	-	-	-	1	-
N. discreta [?]	?	0	1	-	-	-	-	1	-
N. tetrasperma [?]	?	0	1	-	-	-	-	1	-
S. macrospora [?]	?	0	1	-	-	-	-	1	-
T. melanosporum [?]	?	0	1	-	-	-	-	1	-
S. cryophilus [?]	?	0	1	-	-	-	-	1	-
S. japonicus [?]	?	0	1	-	-	-	-	1	-
S. octosporus [?]	?	0	1	-	-	-	-	1	-
S. pombe [?]	?	0	1	-	-	-	-	1	-

Eurotiomycetes	Compl.	No. Class	No. Seq	1	2	3	4	Or	
E. nidulans [?]	?	0	1	-	-	-	-	1	-
A. carbonarius [?]	?	0	1	-	-	-	-	1	-
A. clavatus [?]	?	0	1	-	-	-	-	1	-
A. flavus [?]	?	0	1	-	-	-	-	1	-
A. niger [?]	?	0	1	-	-	-	-	1	-
A. niger [?]	?	0	1	-	-	-	-	1	-
A. oryzae [?]	?	0	1	-	-	-	-	1	-
A. terreus [?]	?	0	1	-	-	-	-	1	-
P. chrysogenum [?]	?	0	1	-	-	-	-	1	-
P. marneffei [?]	?	0	1	-	-	-	-	1	-
A. fumigatus [?]	?	0	1	-	-	-	-	1	-
A. fumigatus [?]	?	0	1	-	-	-	-	1	-
N. fischeri [?]	?	0	1	-	-	-	-	1	-

T. stipitatus [?]	?	0	1	-	-	-	-	1	-
A. capsulatus [?]	?	0	1	-	-	-	-	1	-
A. capsulatus [?]	?	0	1	-	-	-	-	1	-
A. capsulatus [?]	?	0	1	-	-	-	-	1	-
A. capsulatus [?]	?	0	1	-	-	-	-	1	-
A. capsulatus [?]	?	0	1	-	-	-	-	1	-
A. dermatitidis [?]	?	0	1	-	-	-	-	1	-
A. dermatitidis [?]	?	0	1	-	-	-	-	1	-
A. benhamiae [?]	?	0	1	-	-	-	-	1	-
A. gypseum [?]	?	0	1	-	-	-	-	1	-
A. otae [?]	?	0	1	-	-	-	-	1	-
T. equinum [?]	?	0	1	-	-	-	-	1	-
T. rubrum [?]	?	0	1	-	-	-	-	1	-
T. tonsurans [?]	?	0	1	-	-	-	-	1	-
T. verrucosum [?]	?	0	1	-	-	-	-	1	-
A. apis [?]	?	0	1	-	-	-	-	1	-
C. immitis [?]	?	0	1	-	-	-	-	1	-
C. immitis [?]	?	0	1	-	-	-	-	1	-
C. immitis [?]	?	0	1	-	-	-	-	1	-
C. immitis [?]	?	0	1	-	-	-	-	1	-
C. posadasii [?]	?	0	1	-	-	-	-	1	-
C. posadasii [?]	?	0	1	-	-	-	-	1	-
P. brasiliensis [?]	?	0	1	-	-	-	-	1	-
P. brasiliensis [?]	?	0	1	-	-	-	-	1	-
P. brasiliensis [?]	?	0	1	-	-	-	-	1	-
U. reesii [?]	?	0	1	-	-	-	-	1	-

Saccharomycetes	Compl.	No. Class	No. Seq	1	2	3	4	Or	
D. hansenii [?]	?	0	1	-	-	-	-	1	-
L. elongisporus [?]	?	0	1	-	-	-	-	1	-
M. guilliermondii [?]	?	0	1	-	-	-	-	1	-
S. stipitis [?]	?	0	1	-	-	-	-	1	-
Y. lipolytica [?]	?	0	1	-	-	-	-	1	-
C. lusitaniae [?]	?	0	1	-	-	-	-	1	-
C. parapsilosis [?]	?	0	1	-	-	-	-	1	-
C. albicans [?]	?	0	1	-	-	-	-	1	-
C. albicans [?]	?	0	1	-	-	-	-	1	-
C. dubliniensis [?]	?	0	1	-	-	-	-	1	-
C. tropicalis [?]	?	0	1	-	-	-	-	1	-
E. gossypii [?]	?	0	1	-	-	-	-	1	-
K. aestuarii [?]	?	0	1	-	-	-	-	1	-
K. lactis [?]	?	0	1	-	-	-	-	1	-
K. wickerhamii [?]	?	0	1	-	-	-	-	1	-
L. kluyveri [?]	?	0	1	-	-	-	-	1	-
L. thermotolerans [?]	?	0	1	-	-	-	-	1	-
L. waltii [?]	?	0	1	-	-	-	-	1	-
C. glabrata [?]	?	0	1	-	-	-	-	1	-
N. castellii [?]	?	0	1	-	-	-	-	1	-
P. angusta [?]	?	0	1	-	-	-	-	1	-
P. pastoris [?]	?	0	1	-	-	-	-	1	-
P. pastoris [?]	?	0	1	-	-	-	-	1	-
S. bayanus [?]	?	0	1	-	-	-	-	1	-

S. bayanus [?]	?	0	1	-	-	-	-	1	-
S. cerevisiae [?]	?	0	1	-	-	-	-	1	-
S. cerevisiae [?]	?	0	1	-	-	-	-	1	-
S. cerevisiae [?]	?	0	1	-	-	-	-	1	-
S. kudriavzevii [?]	?	0	1	-	-	-	-	1	-
S. mikatae [?]	?	0	1	-	-	-	-	1	-
S. paradoxus [?]	?	0	1	-	-	-	-	1	-
V. polyspora [?]	?	0	1	-	-	-	-	1	-
Z. rouxii [?]	?	0	1	-	-	-	-	1	-
W. anomalus [?]	?	0	1	-	-	-	-	1	-

Basidiomycota	Compl.	No. Class	No. Seq	1	2	3	4	Or	
P. chrysosporium [?]	?	0	1	-	-	-	-	1	-
P. strigosozonata [?]	?	0	1	-	-	-	-	1	-
G. trabeum [?]	?	0	1	-	-	-	-	1	-
F. pinicola [?]	?	0	1	-	-	-	-	1	-
G. subvermispora [?]	?	0	1	-	-	-	-	1	-
P. placenta [?]	?	0	3	-	-	-	-	3	-
T. versicolor [?]	?	0	1	-	-	-	-	1	-
W. cocos [?]	?	0	1	-	-	-	-	1	-
D. squalens [?]	?	0	1	-	-	-	-	1	-
H. annosum [?]	?	0	1	-	-	-	-	1	-
S. hirsutum [?]	?	0	1	-	-	-	-	1	-
A. bisporus [?]	?	0	1	-	-	-	-	1	-
A. bisporus [?]	?	0	1	-	-	-	-	1	-
L. edodes [?]	?	0	1	-	-	-	-	1	-
P. ostreatus [?]	?	0	1	-	-	-	-	1	-
C. cinerea [?]	?	0	1	-	-	-	-	1	-
S. commune [?]	?	0	1	-	-	-	-	1	-
L. bicolor [?]	?	0	2	-	-	-	-	2	-
C. puteana [?]	?	0	1	-	-	-	-	1	-
S. lacrymans [?]	?	0	1	-	-	-	-	1	-
F. neoformans [?]	?	1	2	-	-	1	-	1	-
F. neoformans [?]	?	1	1	-	-	1	-	-	-
F. neoformans [?]	?	1	2	-	-	1	-	1	-
F. neoformans [?]	?	1	2	-	-	1	-	1	-
F. neoformans [?]	?	1	2	-	-	1	-	1	-
T. mesenterica [?]	?	0	1	-	-	-	-	1	-
M. violaceum [?]	?	1	2	-	-	1	-	1	-
R. graminis [?]	?	1	2	-	-	1	-	1	-
S. roseus [?]	?	1	2	-	-	1	-	1	-
M. laricis-populina [?]	?	1	2	-	-	1	-	1	-
P. pachyrhizi [?]	?	0	1	-	-	-	-	1	-
P. graminis [?]	?	1	2	-	-	1	-	1	-
P. triticina [?]	?	1	2	-	-	1	-	1	-
M. globosa [?]	?	0	1	-	-	-	-	1	-
U. maydis [?]	?	0	1	-	-	-	-	1	-
U. maydis [?]	?	0	1	-	-	-	-	1	-

Metazoa	Compl.	No. Class	No. Seq	1	2	3	4	Or	
H. microstoma [?]	?	1	2	2	-	-	-	-	-

E. granulosus [?]	?	1	2	2	-	-	-	-	-
E. multilocularis [?]	?	1	2	2	-	-	-	-	-
S. japonicum [?]	?	1	1	1	-	-	-	-	-
S. mansoni [?]	?	1	1	1	-	-	-	-	-
D. japonica [?]	?	1	1	1	-	-	-	-	-
S. mediterranea [?]	?	1	1	1	-	-	-	-	-
S. kowalevskii [?]	?	1	1	1	-	-	-	-	-
H. robusta [?]	?	2	6	4	2	-	-	-	-
C. teleta [?]	?	3	3	1	1	1	-	-	-
N. vectensis [?]	?	3	3	1	1	1	-	-	-
H. magnipapillata [?]	?	1	1	1	-	-	-	-	-
T. adhaerens [?]	?	1	2	-	2	-	-	-	-
A. queenslandica [?]	?	2	2	-	1	1	-	-	-

Chordata	Compl.	No. Class	No. Seq	1	2	3	4	Or	
B. floridae [?]	?	3	3	1	1	1	-	-	-
O. dioica [?]	?	1	1	1	-	-	-	-	-

Actinopterygii	Compl.	No. Class	No. Seq	1	2	3	4	Or	
O. latipes [?]	?	3	9	5	3	1	-	-	-
O. latipes [?]	?	2	5	4	1	-	-	-	-
F. heteroclitus [?]	?	1	2	2	-	-	-	-	-
G. aculeatus [?]	?	3	9	5	3	1	-	-	-
T. rubripes [?]	?	3	9	5	3	1	-	-	-
T. nigroviridis [?]	?	3	9	5	3	1	-	-	-
C. carpio [?]	?	1	1	1	-	-	-	-	-
B. rerio [?]	?	3	10	5	4	1	-	-	-

Mammalia	Compl.	No. Class	No. Seq	1	2	3	4	Or	
O. anatinus [?]	?	3	4	2	1	1	-	-	-
L. africana [?]	?	3	7	4	2	1	-	-	-
E. telfairi [?]	?	1	1	1	-	-	-	-	3
O. cuniculus [?]	?	3	7	4	2	1	-	-	-
T. belangeri [?]	?	1	4	4	-	-	-	-	-
C. lupus [?]	?	3	7	4	2	1	-	-	-
A. melanoleuca [?]	?	3	7	4	2	1	-	-	-
F. catus [?]	?	2	5	4	1	-	-	-	-
B. taurus [?]	?	3	7	4	2	1	-	-	-
S. scrofa [?]	?	2	3	2	1	-	-	-	-
M. lucifugus [?]	?	3	7	4	2	1	-	-	-
E. europaeus [?]	?	1	4	4	-	-	-	-	-
S. araneus [?]	?	2	3	2	1	-	-	-	-
E. caballus [?]	?	3	7	4	2	1	-	-	-
D. novemcinctus [?]	?	1	1	1	-	-	-	-	-
M. domestica [?]	?	3	7	4	2	1	-	-	-

Rodentia	Compl.	No. Class	No. Seq	1	2	3	4	Or	
C. porcellus [?]	?	3	7	4	2	1	-	-	-
M. musculus [?]	?	3	7	4	2	1	-	-	-
R. norvegicus [?]	?	3	7	4	2	1	-	-	-
S. tridecemlineatus [?]	?	2	4	3	1	-	-	-	-

Primates	Compl.	No. Class	No. Seq	1	2	3	4	Or	
M. fascicularis [?]	?	1	1	1	-	-	-	-	-
M. mulatta [?]	?	3	7	4	2	1	-	-	-
P. hamadryas [?]	?	3	7	4	2	1	-	-	-
G. gorilla [?]	?	3	7	4	2	1	-	-	-
H. sapiens [?]	?	3	7	4	2	1	-	-	-
P. troglodytes [?]	?	3	7	4	2	1	-	-	-
P. abelii [?]	?	3	7	4	2	1	-	-	-
C. jacchus [?]	?	3	7	4	2	1	-	-	-
M. murinus [?]	?	1	3	3	-	-	-	-	-
O. garnettii [?]	?	2	5	4	1	-	-	-	-

Aves	Compl.	No. Class	No. Seq	1	2	3	4	Or	
G. gallus [?]	?	3	6	3	2	1	-	-	-
T. guttata [?]	?	3	6	3	2	1	-	-	-

Lepidosauria	Compl.	No. Class	No. Seq	1	2	3	4	Or	
A. carolinensis [?]	?	3	7	4	2	1	-	-	-

Amphibia	Compl.	No. Class	No. Seq	1	2	3	4	Or	
X. tropicalis [?]	?	3	6	3	2	1	-	-	-
X. laevis [?]	?	2	5	3	2	-	-	-	-

Ascidiacea	Compl.	No. Class	No. Seq	1	2	3	4	Or	
C. intestinalis [?]	?	2	2	1	-	1	-	-	-
C. savignyi [?]	?	2	2	1	-	1	-	-	-

Echinodermata	Compl.	No. Class	No. Seq	1	2	3	4	Or	
S. purpuratus [?]	?	3	3	1	1	1	-	-	-

Mollusca	Compl.	No. Class	No. Seq	1	2	3	4	Or	
A. californica [?]	?	1	1	1	-	-	-	-	-
L. gigantea [?]	?	3	3	1	1	1	-	-	-

Arthropoda	Compl.	No. Class	No. Seq	1	2	3	4	Or	
I. scapularis [?]	?	3	3	1	1	1	-	-	-
D. pulex [?]	?	3	4	2	1	1	-	-	-

Insecta	Compl.	No. Class	No. Seq	1	2	3	4	Or	
T. castaneum [?]	?	3	3	1	1	1	-	-	-
A. florea [?]	?	3	3	1	1	1	-	-	-
A. mellifera [?]	?	3	3	1	1	1	-	-	-
B. terrestris [?]	?	3	3	1	1	1	-	-	-
C. floridanus [?]	?	3	3	1	1	1	-	-	-
A. cephalotes [?]	?	3	3	1	1	1	-	-	-
H. saltator [?]	?	3	3	1	1	1	-	-	-
N. vitripennis [?]	?	3	3	1	1	1	-	-	-
R. prolixus [?]	?	3	3	1	1	1	-	-	-

A. pisum [?]	?	2	2	1	1	-	-	-	-
P. humanus [?]	?	2	2	1	-	1	-	-	-

Diptera	Compl.	No. Class	No. Seq	1	2	3	4	Or	
D. mojavensis [?]	?	2	2	1	-	1	-	-	-
D. virilis [?]	?	2	2	1	-	1	-	-	-
D. grimshawi [?]	?	2	2	1	-	1	-	-	-
D. ananassae [?]	?	2	2	1	-	1	-	-	-
D. erecta [?]	?	2	2	1	-	1	-	-	-
D. melanogaster [?]	?	2	2	1	-	1	-	-	-
D. sechellia [?]	?	2	2	1	-	1	-	-	-
D. simulans [?]	?	1	1	1	-	-	-	-	-
D. simulans [?]	?	1	1	1	-	-	-	-	-
D. simulans [?]	?	1	1	1	-	-	-	-	-
D. simulans [?]	?	1	1	-	-	1	-	-	-
D. simulans [?]	?	1	1	1	-	-	-	-	-
D. simulans [?]	?	1	1	1	-	-	-	-	-
D. simulans [?]	?	1	1	1	-	-	-	-	-
D. simulans [?]	?	1	1	1	-	-	-	-	-
D. yakuba [?]	?	2	2	1	-	1	-	-	-
D. persimilis [?]	?	2	3	2	-	1	-	-	-
D. pseudoobscura [?]	?	2	3	2	-	1	-	-	-
D. willistoni [?]	?	2	2	1	-	1	-	-	-
G. morsitans [?]	?	2	2	1	-	1	-	-	-
M. destructor [?]	?	3	3	1	1	1	-	-	-
A. gambiae [?]	?	3	3	1	1	1	-	-	-
A. darlingi [?]	?	3	3	1	1	1	-	-	-
A. aegypti [?]	?	3	3	1	1	1	-	-	-
C. pipiens [?]	?	3	3	1	1	1	-	-	-

Nematoda	Compl.	No. Class	No. Seq	1	2	3	4	Or	
P. pacificus [?]	?	2	2	1	-	1	-	-	-
B. malayi [?]	?	2	2	1	1	-	-	-	-
L. loa [?]	?	2	2	1	1	-	-	-	-
O. volvulus [?]	?	2	2	1	1	-	-	-	-
W. bancrofti [?]	?	2	2	1	1	-	-	-	-
G. pallida [?]	?	1	1	1	-	-	-	-	-
H. glycines [?]	?	1	1	1	-	-	-	-	-
M. hapla [?]	?	1	1	1	-	-	-	-	-
M. incognita [?]	?	1	1	1	-	-	-	-	-
T. spiralis [?]	?	2	2	1	1	-	-	-	-

Rhabditida	Compl.	No. Class	No. Seq	1	2	3	4	Or	
S. ratti [?]	?	2	2	1	-	1	-	-	-
H. bacteriophora [?]	?	2	2	1	-	1	-	-	-
C. brenneri [?]	?	2	2	1	-	1	-	-	-
C. briggsae [?]	?	2	2	1	-	1	-	-	-
C. elegans [?]	?	2	2	1	-	1	-	-	-
C. japonica [?]	?	2	2	1	-	1	-	-	-
C. remanei [?]	?	2	2	1	-	1	-	-	-

stramenopiles	Compl.	No. Class	No. Seq	1	2	3	4	Or	
F. cylindrus [?]	?	0	1	-	-	-	-	1	-
P. tricornutum [?]	?	0	1	-	-	-	-	1	-
T. pseudonana [?]	?	0	1	-	-	-	-	1	-
B. hominis [?]	?	0	2	-	-	-	-	2	-
A. laibachii [?]	?	0	1	-	-	-	-	1	-
H. arabidopsidis [?]	?	1	2	-	-	1	-	1	3
P. capsici [?]	?	0	1	-	-	-	-	1	-
P. ramorum [?]	?	1	2	-	-	1	-	1	-
P. sojae [?]	?	1	2	-	-	1	-	1	-
P. infestans [?]	?	1	2	-	-	1	-	1	-
P. ultimum [?]	?	1	2	-	-	1	-	1	-
S. parasitica [?]	?	1	2	-	-	1	-	1	-
A. anophagefferens [?]	?	1	3	-	-	1	-	2	-
E. siliculosus [?]	?	1	2	-	-	1	-	1	-
