# Supplementary material for: A holistic phylogeny of the coronin gene family reveals an ancient origin of the tandem-coronin, defines a new subfamily, and predicts protein function
Source: BMC Evol Biol. 2011 Sep 25;11:268. doi: 10.1186/1471-2148-11-268 (PMC3203266; doi:10.1186/1471-2148-11-268)
Supplement: Additional file 5 — Conserved residues in the coronin domain This figure contains the sequence conservation of the entire coronin domain including all mutagenesis experiments as described in Cai et al. [40] and Gandhi et al. [41]. [file 1471-2148-11-268-S5.PDF]

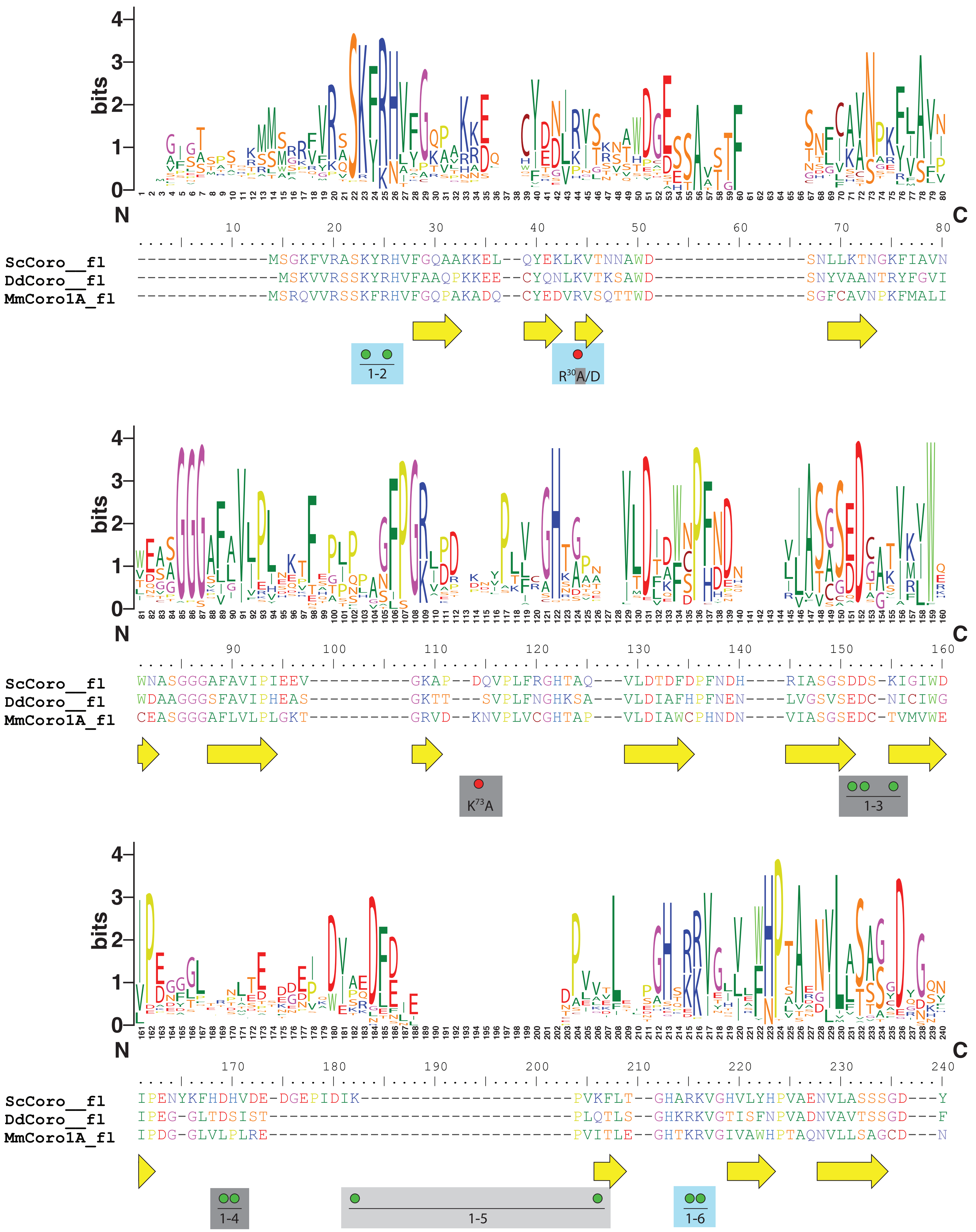

bits
N
C
ScCoro__fl -------------MSGKFVRASKYRHVFGQAAKKEL--QYEKLKVTNNAWD---------------SNLLKTNGKFIAVN
DdCoro__fl --------------MSKVVRSSKYRHVFAAQPKKEE--CYQNLKVTKSAWD---------------SNYVAANTRYFGVI
MmCoro1A_fl -------------MSRQVVRSSKFRHVFGQPAKADQ--CYEDVRVSQTTWD---------------SGFCAVNPKFMALI
1-2
R30A/D
ScCoro__fl WNASGGGAFAVIPIEEV----------GKAP--DQVPLFRGHTAQ---VLDTDFDPFNDH----RIASGSDDS-KIGIWD
DdCoro__fl WDAAGGGSFAVIPHEAS-----------GKTT---SVPLFNGHKSA---VLDIAFHPFNEN----LVGSVSEDC-NICIWG
MmCoro1A_fl CEASGGGAFLVLPLGKT-----------GRVD--KNVPLVCGHTAP---VLDIAWCPHNDN----VIASGSEDC-TVMVWE
K73A
1-3
ScCoro__fl IPENYKFHDHVDE-DGEPIDIK--------------------PVKFLT--GHARKVGHVLYHPVAENVLASSSGD---Y
DdCoro__fl IPEG-GLTDSIST------------------------------PLQTLS--GHKRKVGTISFNPVADNVAVTSSGD---F
MmCoro1A_fl IPDG-GLVLPLRE------------------------------PVITLE--GHTKRVGIVAWHPTAQNVLLSAGCD---N
1-4
1-5
1-6

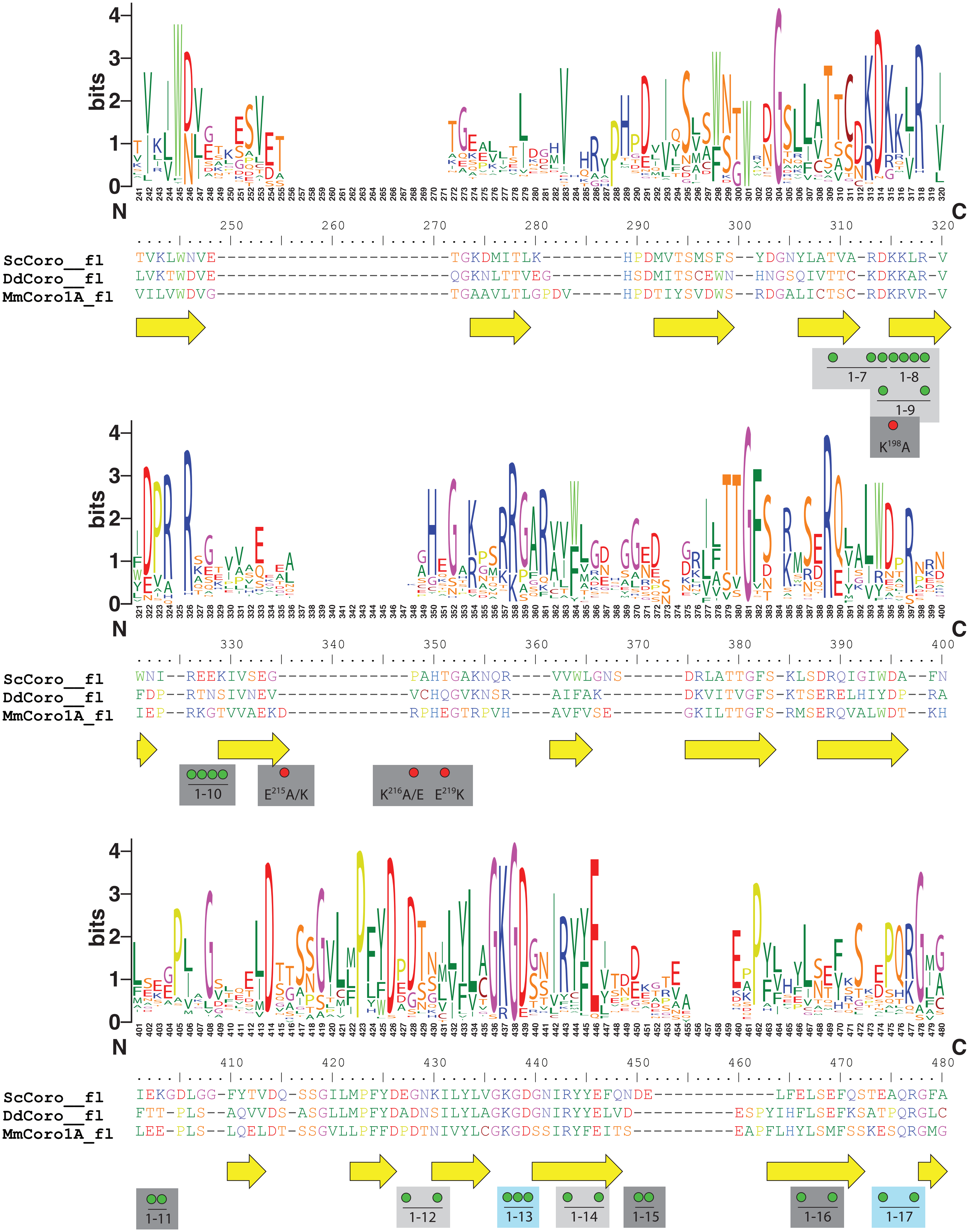
bits
N
C
250 260 270 280 290 300 310 320
ScCoro__fl TVKLWNVE------------------------TGKDMITLK--------HPDMVTSMSFS--YDGNYLATVA-RDKKLR-V
DdCoro__fl LVKTWDVE------------------------QGKNLTTVEG-------HSDMITSCEWN--HNGSQIVTTC-KDKKAR-V
MmCoro1A_fl VILVWDVG------------------------TGAAVLTLGPDV-----HPDTIYSVDWS--RDGALICTSC-RDKRVR-V
1-7
1-8
1-9
K198A
bits
N
C
330 340 350 360 370 380 390 400
ScCoro__fl WNI--REEKIVSEG-------------PAHTGAKNQR----VVWLGNS------DRLATTGFS-KLSDRQIGIWDA--FN
DdCoro__fl FDP--RTNSIVNEV-------------VCHQGVKNSR----AIFAK--------DKVITVGFS-KTSERELHIYDP--RA
MmCoro1A_fl IEP--RKGTVVAEKD------------RPHEGTRPVH----AVFVSE-------GKILTTGFS-RMSERQVALWDT--KH
1-10
E215A/K
K216A/E E219K
bits
N
C
410 420 430 440 450 460 470 480
ScCoro__fl IEKGDLGG-FYTVDQ-SSGILMPFYDEGNKILYLVGKGDGNIRYYEFQNDE------------LFELSEFQSTEAQRGFA
DdCoro__fl FTT-PLS--AQVVDS-ASGLLMPFYDADNSILYLAGKGDGNIRYYELVD----------ESPYIHFLSEFKSATPQRGLC
MmCoro1A_fl LEE-PLS--LQELDT-SSGVLLPFFDPDTNIVYLCGKGDSSIRYFEITS----------EAPFLHYLSMFSSKESQRGMG
1-11
1-12
1-13
1-14
1-15
1-16
1-17

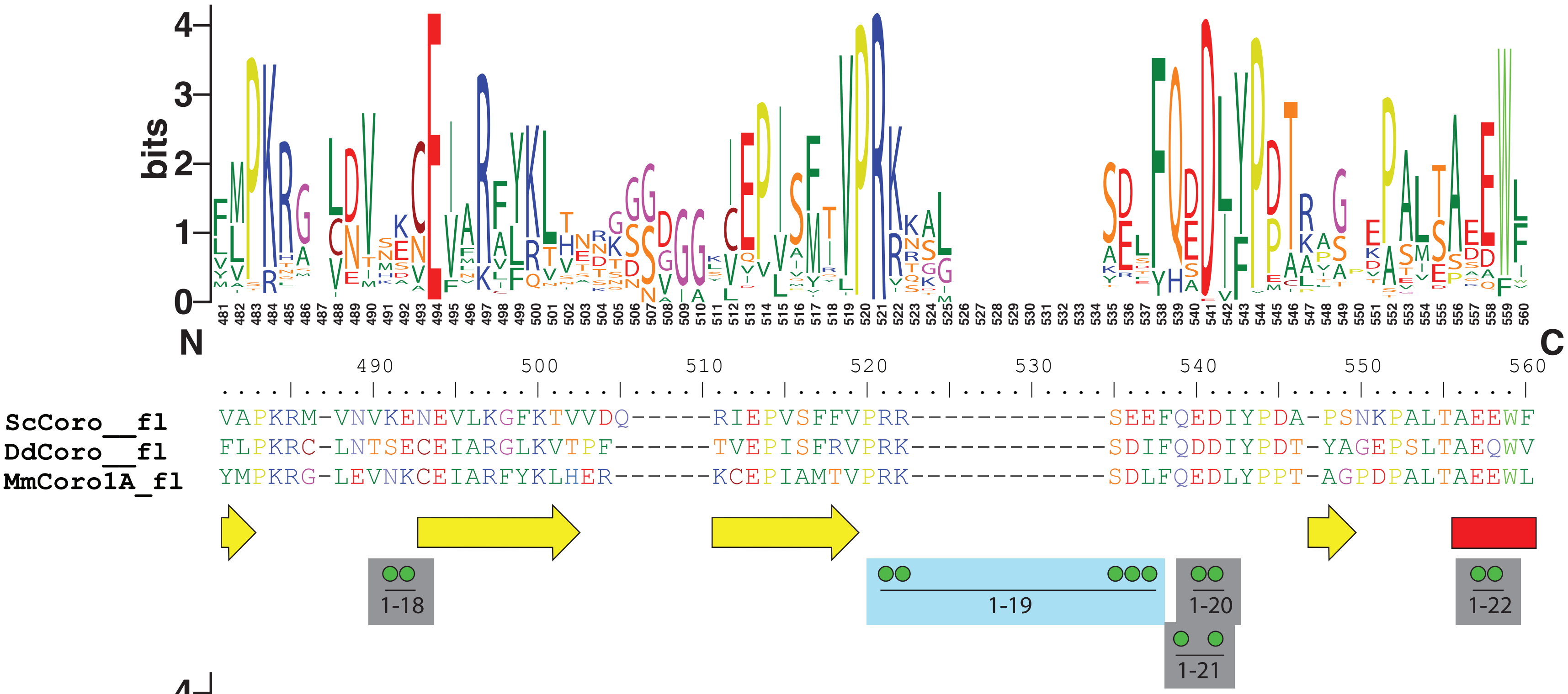

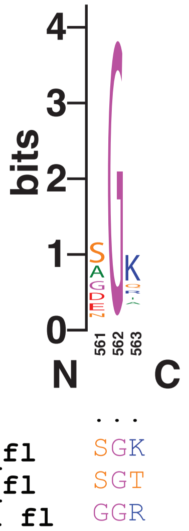

🔴 *Amino acid of HsCoro1A that had been mutated* [L. Cai *et al.* J Cell Sci **2007**, 120, 1779-1790]

🟢 *Amino acid of ScCoro that had been mutated to alanine* [M. Gandhi *et al.* J BiolChem **2010**, 285, 34899-34908]

Alanine and charge-reversal mutagenesis abolishing actin binding

Alanine and charge-reversal mutagenesis not impairing actin binding

Alanine mutants of ScCoro could not be expressed and tested
